# Supplementary material for: Germline thymidylate synthase deficiency impacts nucleotide metabolism and causes dyskeratosis congenita
Source: Am J Hum Genet. 2022 Aug 5;109(8):1472–83. doi: 10.1016/j.ajhg.2022.06.014 (PMC9388389; doi:10.1016/j.ajhg.2022.06.014)
Supplement: Document S1. Tables S1–S3 and Figures S1–S7 [file mmc1.pdf]

The American Journal of Human Genetics, Volume *109*

Supplemental information

Germline thymidylate synthase deficiency impacts nucleotide metabolism and causes dyskeratosis congenita

Hemanth Tummala, Amanda Walne, Roberto Buccafusca, Jenna Alnajar, Anita Szabo, Peter Robinson, Allyn McConkie-Rosell, Meredith Wilson, Suzanne Crowley, Veronica Kinsler, Anna-Maria Ewins, Pradeepa M. Madapura, Manthan Patel, Nikolas Pontikos, Veryan Codd, Tom Vulliamy, and Inderjeet Dokal

Supplementary Information

Table S1: Antibodies used in the study

Antibody	Company	Catalogue number	Dilution
TYMS	Abcam	ab108995	WB 1:2000
TYMK (TK1)	Cell signalling	8960S	WB 1:1000
RRM1	antibodies-online GmbH	ABIN2150979	WB 1:1000
RRM2	Insight Biotechnology	GTX124441-S	WB 1:1000
HSP70	Enzo life sciences	ADI-SPA-819-D	CoIP 1:500
HSP70	Insight Biotechnology	SAB-40416-1	CoIP 1:500
NDPK	antibodies-online GmbH	ABIN5547672	WB 1:1000
β Actin	Abcam	ab8227	WB 1:3000
α tubulin	Abcam	ab7291	WB 1:3000
GAPDH	Abcam	ab8245	WB 1:3000
ATM	Abcam	ab78	WB 1:1000
Phospho-ATM Serine 1981	Abcam	ab208775	WB 1:1000
CHK1	Abcam	ab79758	WB 1:1000
Phospho-CHK1 Serine 345	Abcam	ab58567	WB 1:1000
CHK2	Abcam	ab109413	WB 1:1000
Phospho-CHK2 Threonine 68	Abcam	ab32418	WB 1:1000
TP53	Cell signalling	48818S	WB 1:1000
Phospho p53 Serine 15	Cell signalling	9284T	WB 1:1000
P21	Abcam	AB109520	WB 1:500
DKC1	Abcam	EPR10398	WB 1:1000
TERT	Abcam	ab32020	WB 1:1000
SMUG	Abcam	ab192240	WB 1:1000
TIN2	Abcam	ab197894	WB 1:1000
POT1	Enzo life sciences	PSC-15-787-R050	WB 1:1000
TPP1	antibodies-online GmbH	ABIN5074829	WB 1:1000
TRF1	Insight Biotechnology	TA309899	WB 1:1000
H2AX serine 139	Cell signalling	9718S	IF 1:300

Source of antibody procurement and relevant dilutions used in experiments. WB refers to western blot and ICC refers to immuno cyto chemistry.

Table S2: Bone marrow failure gene panel

ACD	G6PC3	RPL5
ADA2	GATA1	RPL9
ANKRD26	GATA2	RPS10
BRCA1	GFI1	RPS17
BRCA2	GRHL2	RPS19
BRIP1	HAX1	RPS24
C15orf41	HOXA11	RPS26
CDAN1	JAGN1	RPS27
CEBPA	KIF23	RPS28
CSF3R	KLF1	RPS29
CTC1	LIG4	RPS7
CXCR4	LPIN2	RTEL1
CYCS	MAD2L2	RUNX1
DCLRE1B	MECOM	SAMD9
DDX41	MPL	SAMD9L
DKC1	MYSM1	SBDS
DNAJC21	NAF1	SEC23B
DNAJC3	NHP2	SHQ1
DUT	NOP10	SLX4
EFL1	NPM1	SP1
ELANE	PALB2	SRP54
ERBB3	PARN	SRP72
ERCC4	POLA1	STN1
ERCC6L2	POT1	TAZ
ETV6	RAD51	TERC
FANCA	RAD51C	TERT
FANCB	RBM8A	THPO
FANCC	RECQL4	TINF2
FANCD2	RFWD3	TP53
FANCE	RMRP	TYMS
FANCF	RPL11	UBE2T
FANCG	RPL15	USB1
FANCI	RPL18	VPS45
FANCL	RPL26	WAS
FANCM	RPL27	WRAP53
FYB1	RPL31	XRCC2
G6PC	RPL35A	ZCCHC8

Table S3: Variants in *TYMS*- *ENOSF1* locus

Family	Individual	*TYMS* Variant	Additional variant (NC_000018.10: g.623000-716000)	gnomAD MAF	Location
1	father	.	.	.	.
1	Index/proband (M)	c.485_487delAA: pR163SfsTer3	.	.	.
1	mother	c.485_487delAA: pR163SfsTer3	.	.	.
2	father	c.485_487delAA: pR163SfsTer3	.	.	.
2	Index/proband (M)	c.485_487delAA: pR163SfsTer3	NC_000018.10:g.706815A>T (LCR)	NR	*ENOSF1*, intron 1
2	mother	.	NC_000018.10:g.706815A>T (LCR)	NR	*ENOSF1*, intron 1
2	U/A brother	.	.	.	.
3	father	c.343C>T: p.R115X	.	.	.
3	Index/proband (M)	c.343C>T: p.R115X	NC_000018.10:g.694842T>C	0.0009	*ENOSF1*, intron 3
3	mother	.	NC_000018.10:g.694842T>C	0.0009	*ENOSF1*, intron 3
4	affected sister	c.534_535insTG: p.M179X	NC_000018.10:g.694842T>C	0.00026	TYMSOS, intron 1
4	father	c.534_535insTG: p.M179X	.	.	.
4	Index/proband (M)	c.534_535insTG: p.M179X	NC_000018.10:g.654556C>T	0.00026	*TYMSOS*, intron 1
4	mother	.	NC_000018.10:g.654556C>T	0.00026	*TYMSOS*, intron 1
5	father	c.A480T: p.Q160H	.	.	.
5	Index/proband (F)	c.A480T: p.Q160H	NC_000018.10:g.696402C>T	0.0013	*ENOSF1*, intron 3
5	mother	.	NC_000018.10:g.696402C>T	0.0013	*ENOSF1*, intron 3
5	U/A brother	.	.	.	.
6	Index/proband (M)	c.G259A: p.E87K	NC_000018.10:g.694158C>T	NR	*ENOSF1*, intron 4
7	Index/Proband (M)	c.556+1G>A	.	.	.
8	Index/proband (F)	c.C811T: p.R271X	NC_000018.10:g.667547T>A (LCR)	0.0047	*TYMS*, intron 3

U/A – unaffected; MAF- Minor allele frequency; LCR-Low complexity region; M – male; F- female.

Figure S1: Sanger traces of *TYMS* coding sequence from control and proband's whole blood DNA and cDNA

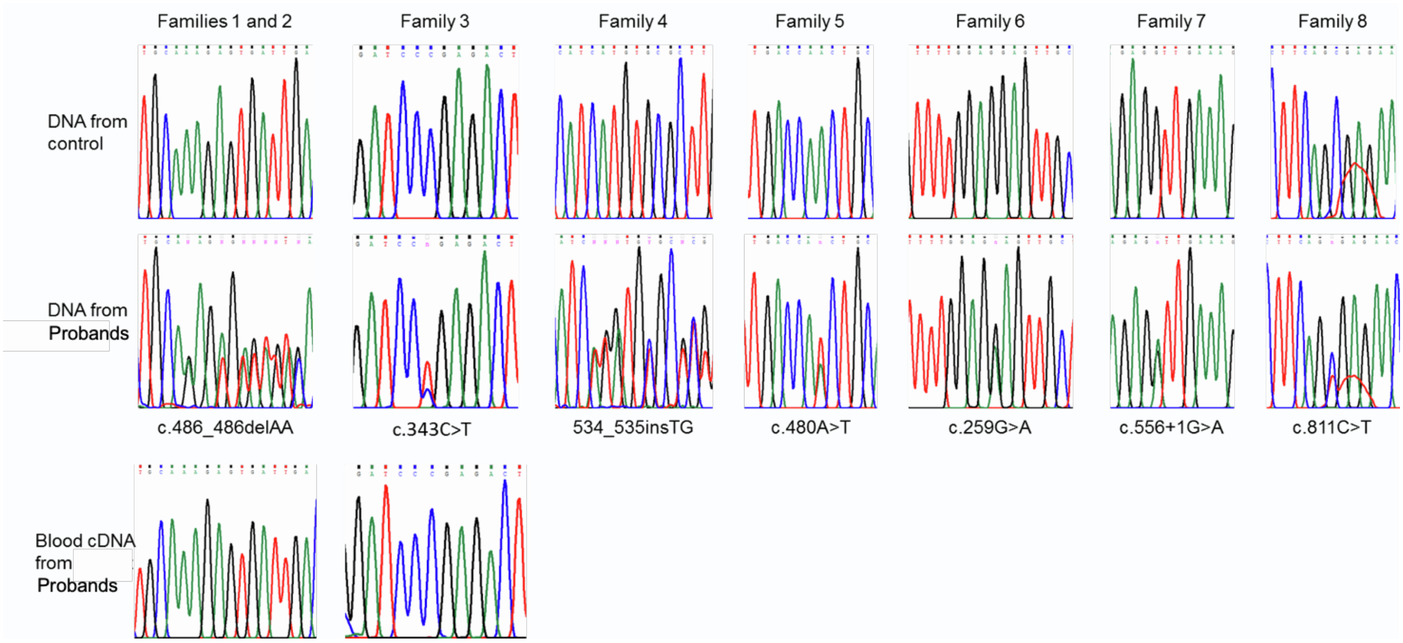

Representative Sanger traces showing the pathogenic variants identified in this study compared to an unaffected sample. Where RNA samples were available from the probands, we show that the variants in Families 1-3 cause nonsense mediated decay of the allele carrying the *TYMS* variant.

Figure S2. Clustal Alignment of *TYMS* in different species

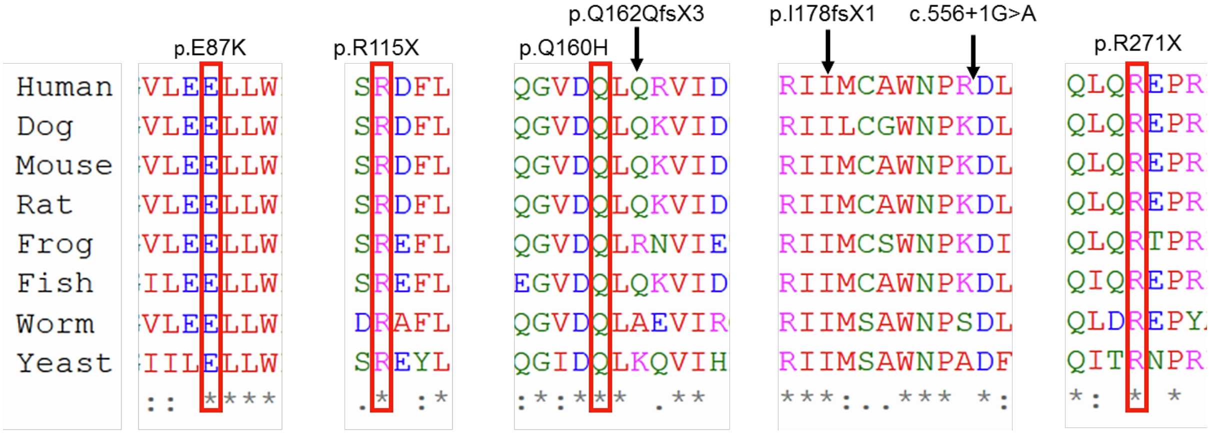

Clustal alignment of TYMS showing a high level of conservation between species. The position of the variants identified in this study are highlighted.

Figure S3: Telomere and telomerase associated growth defects in TYMS deficient cells of affected individuals

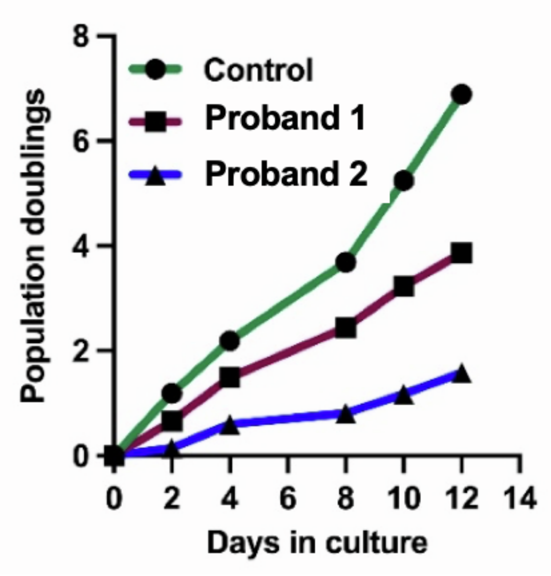

Population doubling rate of probands' cell lines (1 and 2) compared to control.

Figure S4: Assessment of DNA repair proteins in TYMS deficient individual cells along with control cells

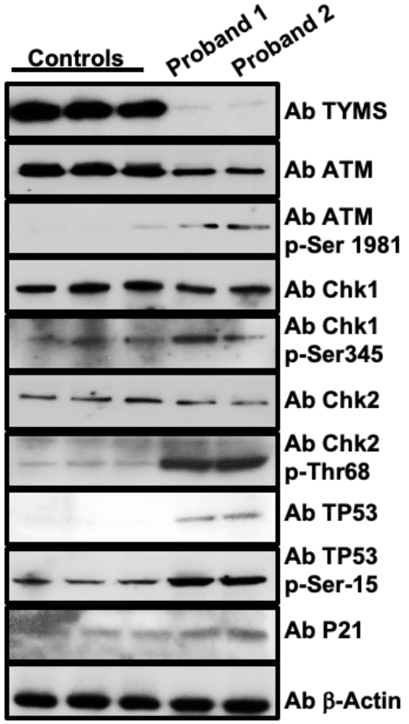

Immunoblotting of several key proteins involved in telomere maintenance and DNA damage response pathway. Antibody against β-Actin is used to determine the loading control.

Figure S5: Post transcriptional fate of *TYMS* is controlled by *ENOSF1*

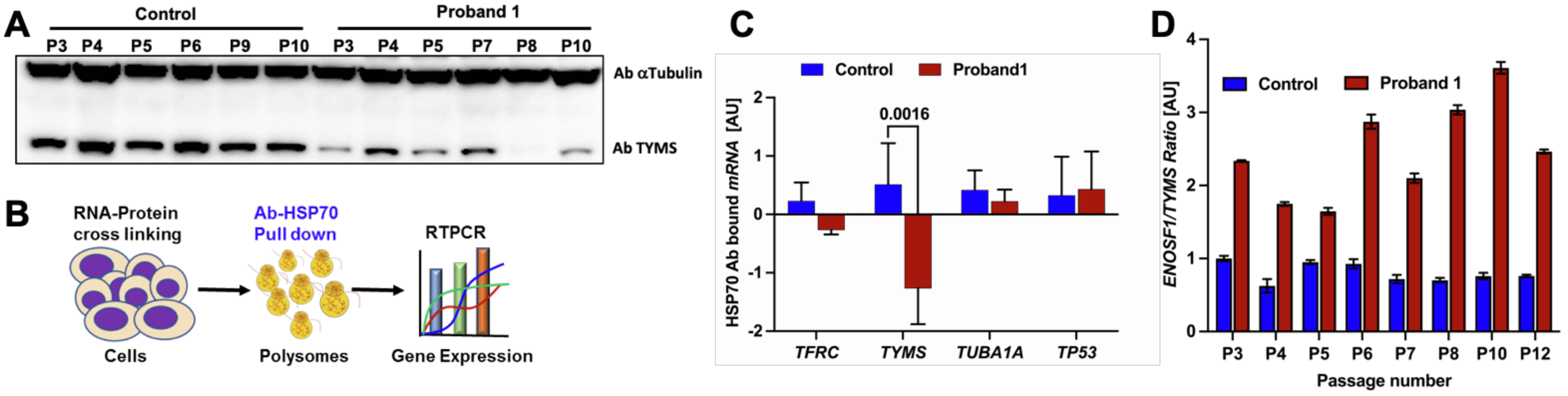

A) Reduced TYMS protein levels in proband samples over time at different passages. Antibody against α-tubulin is used to determine loading control.

B) Schematic diagram of crosslinking and immunoprecipitation of translating ribosome with affinity beads that are covalently attached with anti-Hsp70 antibodies. The crosslinked and anti-Hsp70 immunoprecipitated mRNA complexes are subsequently eluted and analysed by qPCR.

C) qPCR analysis shows a significant decrease in *TYMS* mRNA bound to the polysome when compared to input, while TP53 binding to polysome is increased in the proband samples. Bars represent the median of relative expression ratio.

D) *ENOSF1* transcript levels relative to *TYMS* expression in control and proband cells with increasing passage number.

Figure S6: Endogenous TYMS rescue and RNA-RNA interaction prediction between *TYMS* and *ENOSF1*

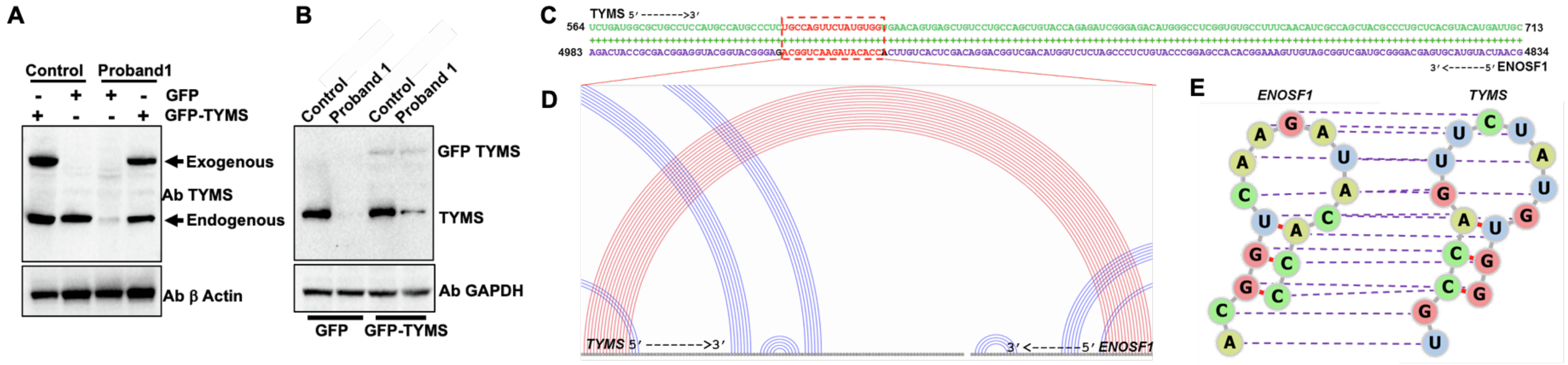

(A and B) Immunoblotting of TYMS protein in control and proband cells in two different transduction experiments at passage 3 (A) and passage 8 (B) transduced with lentivirus particles encoding GFP alone or GFP tagged *TYMS* cDNA. Antibody against β-Actin and α-tubulin is used to determine loading control. (C and D) IntaRNA verison 2.4.1 was used for prediction of RNA sequences involved in mediating RNA-RNA interaction between *TYMS* and *ENOSF1*. The red highlighted region in the sequence shows putative and accurate interaction region involved between *TYMS* and *ENOSF1* using RactIP. The red arched lines indicate intergenic interactions between two different genes, in this proband *TYMS* and *ENOSF1*. The blue arched lines indicated intragenic interaction within the gene.

Figure S7: Clinical features of affected individuals with biallelic *DUT* variants (NM_001025248.2)

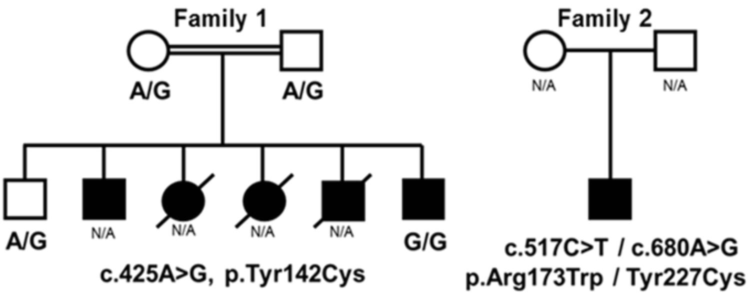

Features of individuals with biallelic *DUT* variants

Family	1	2
**Feature**		
Gender	M	M
Country/ethnic origin	Sudan	UK-Scotland
Age at investigation (years)	5	16
Parents first cousins/related	Y	N
Skin pigmentation abnormalities	Y	Y
Nail dystrophy	N	Y[c]
Leucoplakia	N	N
Hair loss/thin eye lashes	N	N
Haematological abnormalities	Y[a]	Y[d]
Immune defects	N	Y[d]
Learning/developmental difficulties	N	N
Microcephaly	N	N
Other features	Y[b]	Y[e]

Family 1 - (a) Investigations at age 5 years showed pancytopenia, hypocellular bone marrow with dyserythropoiesis and normal karyotype. This individual's history and clinical course have been documented in the European Journal of Haematology (1998; 60: 209-212). He was stable on oxymetholone and granulocyte colony stimulating factor therapy for several years; (b) developed insulin dependent diabetes mellitus while on oxymetholone therapy, at age 10 yrs progressed to myelodysplasia with monosomy 7. His family history was significant, 3 older siblings had died of bone marrow failure associated with diabetes mellitus in Sudan.

Family 2 – (c) many toenails affected; (d) at age 16 years was blood transfusion dependent and lymphopenic. Bone marrow was hypercellular exhibiting marked dyserythropoiesis and associated with splenomegaly; (e) Abnormal facies, small jaw, overcrowded teeth, growth restriction, short stature and hypogonadism. F = female, M = male; Y = yes; N = normal/no
