# Supplementary material for: Germline thymidylate synthase deficiency impacts nucleotide metabolism and causes dyskeratosis congenita
Source: Am J Hum Genet. 2022 Aug 5;109(8):1472–83. doi: 10.1016/j.ajhg.2022.06.014 (PMC9388389; doi:10.1016/j.ajhg.2022.06.014)
Supplement: Document S2. Article plus supplemental information [file mmc2.pdf]

Germline thymidylate synthase deficiency impacts nucleotide metabolism and causes dyskeratosis congenita

Graphical abstract

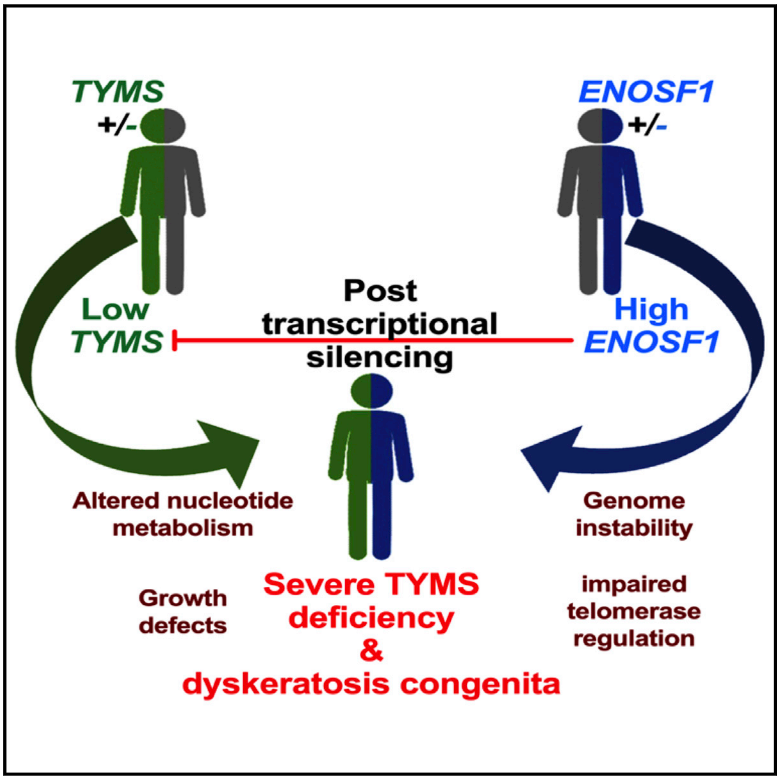

Authors

Hemanth Tummala, Amanda Walne, Roberto Buccafusca, ..., Veryan Codd, Tom Vulliamy, Inderjeet Dokal

Correspondence

h.tummala@qmul.ac.uk

A digenic inheritance pattern of variants occurring at the TYMS-ENOSF1 locus and segregating from parents to proband exert a post-transcriptional epistatic effect in causing severe thymidylate synthase deficiency and disease features of dyskeratosis congenita.

Tummala et al., 2022, The American Journal of Human Genetics *109*, 1472–1483
August 4, 2022 © 2022 The Author(s).
https://doi.org/10.1016/j.ajhg.2022.06.014

CellPress

ARTICLE

Germline thymidylate synthase deficiency impacts nucleotide metabolism and causes dyskeratosis congenita

Hemanth Tummala,[1,12,*] Amanda Walne,[1,12] Roberto Buccafusca,[2] Jenna Alnajar,[1] Anita Szabo,[3] Peter Robinson,[4] Allyn McConkie-Rosell,[5] Meredith Wilson,[6] Suzanne Crowley,[7] Veronica Kinsler,[8] Anna-Maria Ewins,[9] Pradeepa M. Madapura,[1] Manthan Patel,[1] Nikolas Pontikos,[3] Veryan Codd,[10] Tom Vulliamy,[1] and Inderjeet Dokal[1,11]

Summary

Dyskeratosis congenita (DC) is an inherited bone-marrow-failure disorder characterized by a triad of mucocutaneous features that include abnormal skin pigmentation, nail dystrophy, and oral leucoplakia. Despite the identification of several genetic variants that cause DC, a significant proportion of probands remain without a molecular diagnosis. In a cohort of eight independent DC-affected families, we have identified a remarkable series of heterozygous germline variants in the gene encoding thymidylate synthase (*TYMS*). Although the inheritance appeared to be autosomal recessive, one parent in each family had a wild-type *TYMS* coding sequence. Targeted genomic sequencing identified a specific haplotype and rare variants in the naturally occurring TYMS antisense regulator *ENOSF1* (enolase super family 1) inherited from the other parent. Lymphoblastoid cells from affected probands have severe TYMS deficiency, altered cellular deoxyribonucleotide triphosphate pools, and hypersensitivity to the TYMS-specific inhibitor 5-fluorouracil. These defects in the nucleotide metabolism pathway resulted in genotoxic stress, defective transcription, and abnormal telomere maintenance. Gene-rescue studies in cells from affected probands revealed that post-transcriptional epistatic silencing of *TYMS* is occurring via elevated *ENOSF1*. These cell and molecular abnormalities generated by the combination of germline digenic variants at the *TYMS-ENOSF1* locus represent a unique pathogenetic pathway for DC causation in these affected individuals, whereas the parents who are carriers of either of these variants in a singular fashion remain unaffected.

Introduction

A clinically diagnosed individual with dyskeratosis congenita (DC [MIM: 305000]) classically presents with a combination of muco-cutaneous features comprising abnormal skin pigmentation, nail dystrophy, and oral leucoplakia,[1] as initially described by Zinsser in 1910.[2] Bone-marrow failure, predisposition to cancer, and pulmonary abnormalities are reported to be major causes of death in DC-affected individuals. Inheritance of DC can be autosomal dominant, autosomal recessive, or X-linked recessive.

A major subset of individuals (n = 17) with DC have variants in genes that have a principal role in telomere maintenance (*DKC1* [MIM: 300126], *TERC* [MIM: 602322], *TERT* [MIM: 187270], *NOP10* [MIM: 696471], *NHP2* [MIM: 606470], *TINF2* [MIM: 604319], *WRAP53* [MIM: 612661], *CTC1* [MIM: 613129], *RTEL1* [MIM: 608833], *ACD* [MIM: 609377], *PARN* [MIM: 604212], *NAF1* [MIM: 617868], *STN1* [MIM: 6132128], *MDM4* [MIM: 602704], *ZCCHC8* [MIM: 616381], *POT1* [MIM: 606478], and *RPA1* [MIM:179835]).[1] Five of these genes encode components of the enzyme telomerase (*TERC*, telomerase RNA component; TERT, telomerase reverse transcriptase; DKC1, dyskerin pseudouridine synthase 1; NOP10, NOP10 ribonucleoprotein; or NHP2, NHP2 ribonucleoprotein) contributing to its catalytic function. Poly (A)-specific ribonuclease (PARN), zinc finger CCHC-type containing 8 (ZCCHC8), and nuclear assembly factor 1 ribonucleoprotein (NAF1) are involved in *TERC* maturation.[3–5] TERF1-interacting nuclear factor 2 (TIN2), telomerase recruitment factor (ACD, also known as *TPP1*), and protection of telomeres 1 (POT1) are components of the shelterin complex that protects the telomeric DNA and is involved in telomerase recruitment and processivity.[6–8] WD-repeat-containing antisense to TP53 (WRAP53) is important in telomerase trafficking.[9] Telomere replication complex component 1 (CTC1) and STN1 subunit of CST complex (STN1), subunits of the CST-complex-regulated C strand, fill in at telomere ends and further facilitate recruitment and docking of telomerase onto the telomere.[10,11] Regulator of telomere elongation helicase 1 (RTEL1) has a critical role in telomere replication and in dismantling the t loop at telomeres.[12] *MDM4*, which

[1]Genomics and Child Health, Blizard Institute, Queen Mary University of London, Newark Street, London E1 2AT, UK; [2]School of Physical and Chemical Sciences, Queen Mary University of London, Mile End, London E1 4NS, UK; [3]Institute of Ophthalmology, Faculty of Brain Sciences, University College London, 11-43 Bath St, London EC1V 9EL, UK; [4]The Jackson Laboratory for Genomic Medicine, 10 Discovery Dr., Farmington, CT 06032, USA; [5]Division of Medical Genetics, Duke University Medical Center, USA; [6]Department of Clinical Genetics, The Children's Hospital at Westmead, Sydney, Australia; [7]Department of Paediatrics, St George's Healthcare NHS Trust, London, UK; [8]Department of Paediatric Dermatology, Great Ormond Street Hospital, The Francis Crick Institute, London, UK; [9]Haematology/Oncology Department, Royal Hospital for Sick Children, Glasgow, UK; [10]Department of Cardiovascular Sciences, University of Leicester, Leicester, UK; [11]Barts Health NHS Trust, London, UK
[12]These authors contributed equally
*Correspondence: h.tummala@qmul.ac.uk
https://doi.org/10.1016/j.ajhg.2022.06.014.
© 2022 The Author(s). This is an open access article under the CC BY license (http://creativecommons.org/licenses/by/4.0/).

encodes a major negative regulator of P53 protein, has transcriptional control of genes involved in telomere biology.[13] Replication protein A 1(RPA1) is the largest subunit of the DNA replication protein complex that binds to single-stranded DNA and facilitates protein-protein interactions during DNA replication.[14] Although the major molecular function of these genes is in telomere maintenance, it is important to recognize their multifunctional roles. For example, dyskerin, NOP10, NHP2, PARN, and NAF1 are all involved in modification and processing of ribosomal RNA for ribosome biogenesis.

In a subset of individuals with DC, germline defects are observed in genes whose major role is not in telomere maintenance. This includes germline nucleophosmin 1 (NPM1 [MIM: 164040]) variants that affect 2′-O-methylation of rRNA and modulate translation[15] and U6 small nuclear RNA biogenesis phosphodiesterase 1 USB1 [MIM: 613276] that is involved in oligouridylation of U6 small nuclear RNA.[16,17] Given the considerable heterogeneity in the disease spectrum, the uncharacterized DC individuals held in the Dyskeratosis Congenita Registry in London might harbour constitutional mutations or variants in several genes. Here we report the first evidence of a digenic inheritance pattern in a series of individuals who come from eight unrelated families and have homogeneous DC features caused by germline variants in the *TYMS-ENOSF1* locus.

Material and Methods

Sequencing and bioinformatics

Genomic DNA was extracted from peripheral blood samples (Puregene, Qiagen). Exome data were processed and called jointly with a set of 2,500 whole-exome-sequenced internal control samples (UCL-ex consortium) and hg19 as a reference index (UCSC browser) according to the recommendations from the Genome Analysis Toolkit (GATK v3.2) to minimize artefactual batch effects.[18] All variants identified were validated by Sanger sequencing. We then targeted the genomic region corresponding to chr18: 623000–716000 by using the Cell3 Target (Nonacus) custom kit to search for any additional variants that were not detected by the exome capture. The resultant targeted fragments were then sequenced on the Illumina MiSeq platform. Read alignment, variant calling, and annotation were performed with an in-house pipeline involving the Burrows Wheeler aligner, the Genome Analysis Tool kit, and ANNOVAR, respectively. We used IntaRNA version 2.4.1[19] to predict RNA-RNA interaction by using the computational pipeline previously described, and the accurate 2D interaction prediction was performed with RactIP.[20]

Cell culture and treatments

HeLa cells were cultured in Dulbecco's modified Eagle's medium (DMEM), and Epstein-Barr-virus-transformed lymphoblastoid lines (LCLs) were acquired from affected and unaffected individual blood samples and grown in RPMI1640. All culture media were supplemented with 10% (v/v) fetal bovine serum (FBS; HyClone),100 IU/mL penicillin, and 100 mg/mL streptomycin (Invitrogen). Cells were maintained at 37°C in a humidified incubator with 5% CO_2. Cell cultures were supplemented with 5-flurouracil and hydroxyurea over a specified dose range (Sigma-Aldrich). After a 24 h incubation at 37°C, tetrazolium salt WST-1 dye (Merck Millipore) was added to the cell suspension, and the cells were analyzed on a plate reader at 450 nM absorbance (Becton Dickinson). The lentiviral induction was performed with cDNAs encoding *GFP*, *GFP*-tagged *TYMS* (Origene), and *ENOSF1* shRNA (Santa Cruz Biotechnology), in control and probands' cells by spinfection in the presence of polybrene (10 μg/mL). 8 h after transduction, media were replaced, and cells were processed for downstream analysis 24 h later.

Immunoblotting and immunocytochemistry

We prepared protein extracts by lysing washed cells in denaturing buffer (9 M urea, 150 mM 2-mercaptoethanol, and 50 mM Tris-HCl [pH 7.3]) and subsequently sonicating them to shear genomic DNA. Total and phosphorylated forms of ATM, ATR, and DNA-PK were separated on 3%–8% Tris glycine mini gels (Life Technologies). For all other proteins, 4%–12% NuPAGE Bis-Tris mini gels (Life Technologies) were used. Gels were transferred onto PVDF membrane (GE Healthcare). Blotting was performed with primary antibodies (Table S1), and the corresponding alkaline-phosphatase-conjugated secondary antibodies were supplied in the WesternBreeze chemiluminescent kit (Thermo Fisher). α−tubulin, β-actin, and GAPDH antibodies were used as a loading control. For immunocytochemistry, normal and proband cells were subjected to cytospin on poly-D-lysine-coated slides (Sigma), fixed with 4% PFA, and permeabilized with 0.1% Triton X-100 (TX100) in PBS. Fixed cells were quenched in 50 mM NH_4Cl and blocked in 10% goat serum and 1% BSA in PBS containing 0.05% TX100 for 1 h. Cells were incubated with γ-H2AX primary antibody followed by Alexa-Fluor-488-conjugated secondary antibody (Invitrogen) in blocking solution. Cells were washed three times in PBS containing 0.05% TX100 between primary and secondary antibody incubations and mounted with Vectashield containing DAPI (Vector Labs). A Zeiss LSM700 confocal microscope with ZEN software was used, and 63× captured images were acquired.

Quantification of mRNA levels by RT-PCR

RNA was extracted from either whole blood or EBV-transformed LCLs. cDNA was prepared from total RNA by the use of Invitrogen Superscript IV according to manufacturer's instructions with 600 ng input RNA from LCLs and 500 ng input RNA from blood and was primed with an equal mix of anchored dT oligonucleotides and random hexamers unless stated otherwise. A pool of random control cDNAs was prepared and serially diluted to form a relative standard curve against which all samples were quantified. TaqMan probes used were *TYMS* (Hs00426586_m1), *ENOSF1* (Hs01106532_m1), *TFRC* (Hs00951083_m1), *MCM6* (Hs00962418_m1), *TUBA1A* (Hs00362387_m1), and *TP53* (Hs01034249_m1). All reactions were setup with TaqMan Fast Advanced Master Mix according to the manufacturer's instructions. For measuring *TERC* expression, KiCqStart primers (Merck) for *TERC*, *ABL*, *ACTB*, and *GAPDH* were used. The mature TERC pool was assessed in RNA extracted from LCLs and blood. In brief, separate cDNA pools were synthesized with anchored $dT_{(20)}$ oligonucleotides and random hexamers from the same sample. The mature *TERC* pools were calculated by comparison of the amounts of amplified *TERC* between these samples. Four replicates per sample were run on the Roche Lightcycler 480 system. All primers had an amplification efficiency between 90% and 100%.

Each gene of interest (GOI) was normalized against the control gene(s), and the relative (n-fold) change between probands and controls was calculated unless stated otherwise.

Isolation of polysome-associated mRNA

This protocol was adapted as previously described[21] and modified accordingly. In brief, we prepared the cytoplasmic extracts by harvesting proband and control cells in ice-cold PBS containing 100 μg/mL cycloheximide (Sigma). Cells were counted, and 100,000 cells were incubated with 800 μL of RPMI medium containing 10% fetal bovine serum and 100 μg/mL cycloheximide (Sigma) for 5 min at 37°C. After incubation, 200 μL of N-hydroxysuccimide ester (DSP; 1 mM; Pierce) was introduced as a cross-linking reagent and incubated for 5 min at 37°C followed by quenching with 1 M Tris–HCl (pH 7.4). The cells were washed twice by centrifugation at 1,000 r.p.m. for 3 min and rinsed with ice-cold PBS containing 100 μg/mL cycloheximide (Sigma). The final pellets were swollen for 20 min in 500 μL of low-salt buffer (LSB) (20 mM HEPES [pH 7.4], 100 mM KCl, and 2 mM $MgCl_2$) containing 1 mM dithiothreitol and lysed by the addition of 500 μL lysis buffer (1× LSB containing 1.2% Triton X-100) (Sigma) followed by brief vortexing. One-tenth (70 μL) of the above lysate was transferred to the Ig-coated beads, and incubation was carried out for 2 h at 4°C. After incubation with the HSP70/HSP73 antibody-conjugated magnetic beads, the polysome complexes containing translationally active mRNA transcripts were isolated and eluted from beads with the Array Pure Nanoscale RNA Purification Kit (Epicentre).

Telomere length measurement

Telomere lengths were measured either by the monochrome multiplex quantitative PCR (MMQPCR) method modified from Cawthon[22] or by FLOWFISH as supplied by Repeat Diagnostics.[23] For MMQPCR, in each well the amplified telomeric DNA (T) and a single-copy gene (S) were quantified against a standard curve obtained from the dilution of a reference DNA sample. The T/S ratio, obtained in triplicate for each sample, is directly proportional to the telomere length. This ratio was normalized to the T/S ratio of a second reference sample that was run on every plate to give a relative T/S ratio.

Cellular dNTP analysis

In brief, 10^6 cells were extracted with 1 mL of ice-cold 60% methanol at 20°C, followed by centrifugation at 16,000 g for 30 min. The supernatant was heat inactivated and vacuum-dried, then rehydrated in 80 mL of water for cellular dNTP measurement by mass spectrometry as described previously.[24]

Cell-sensitivity assays

Lymphoblastoid cells from affected probands (family 1 II-2; family 2 II-2; and family 3 II-1), parents, and controls were treated with serial doses of either 5-fluorouracil (5-FU) or hydroxyurea dissolved in DMSO at the indicated concentrations. Cell viability was assessed via neutral red-dye uptake by live cells. All chemicals were obtained from Enzo Life Sciences and Sigma Aldrich. All readings were normalized to the untreated sample. We calculated statistical significance by comparing the linear regression of the curves with GraphPad Prism 9 software.

TRAP assay

Cells (10^5) were lysed in CHAPS buffer for 30 min at a concentration of 3,000 cells/μL. The lysates were centrifuged at 14,000 rpm for 20 min at 4°C. We then diluted 2 μL of eluate in 50 μL of PCR-TRAP reaction and subsequently performed quantification by running the PCR products in non-denaturing 20% TBE gels at 120 V for 30 min and then stained them with SYBR Gold nucleic acid gel stain (Thermo Fischer).

Statistical analysis

Statistical analysis was performed with GraphPad Prism software (version 9), and a p value <0.05 was considered statistically significant. In line graphs, for each experimental dataset, we conducted a linear regression to determine the best-fit line describing the data from each independent experiment. The overall significance of cytotoxicity was determined with a one-way ANOVA and post-hoc Tukey's test on the slopes of the regression lines from each data set ($n = 3$ independent experiments performed in octuplicate). In scattered dot plots and bar graphs, a Mann-Whitney test was used for determining significant differences in between different cell lines.

Study approval

The affected individuals and families included in this study had all been recruited to the London Dyskeratosis Congenita registry. Blood samples and clinical information were collected at enrolment. All participants and their family members provided written informed consent in accordance with the declaration of Helsinki and the approval of our local research ethics committee (London [city and east] reference number 07/Q0603/5).

Results

Thymidylate synthase (TYMS) deficiency in affected individuals with germline *TYMS* variants

Recent genome-wide association studies on distinct ethnic human populations have indicated several genes that are important regulators of telomere length.[25–28] We compiled a list of genes that were common to at least two of these studies and did not include any previously identified telomere-associated genes. We analyzed exomes of 189 DC probands for variants in these genes and identified six previously unreported heterozygous variants in the thymidylate synthase (*TYMS* [MIM: 188350; GenBank: NM_001071.4]) in affected probands from six independent families (Figure 1A); four of these variants were loss of function (LOF). Targeted sequencing of two additional families who had a strong phenotypic resemblance to the other families revealed that they also had LOF *TYMS* variants, one of which was seen in a previous family. This series of six LOF variants is a highly significant finding, given that there are only four LOF *TYMS* variants in the gnomAD database, which comprises more than 125,000 individuals ($p < 0.000001$: Chi squared with Yates' correction). None of these individuals harboured unique variants in DC or DC-related genes as identified by exome analysis or a targeted gene panel that comprises 111 genes with known associations with bone-marrow failure (Table S2). What was

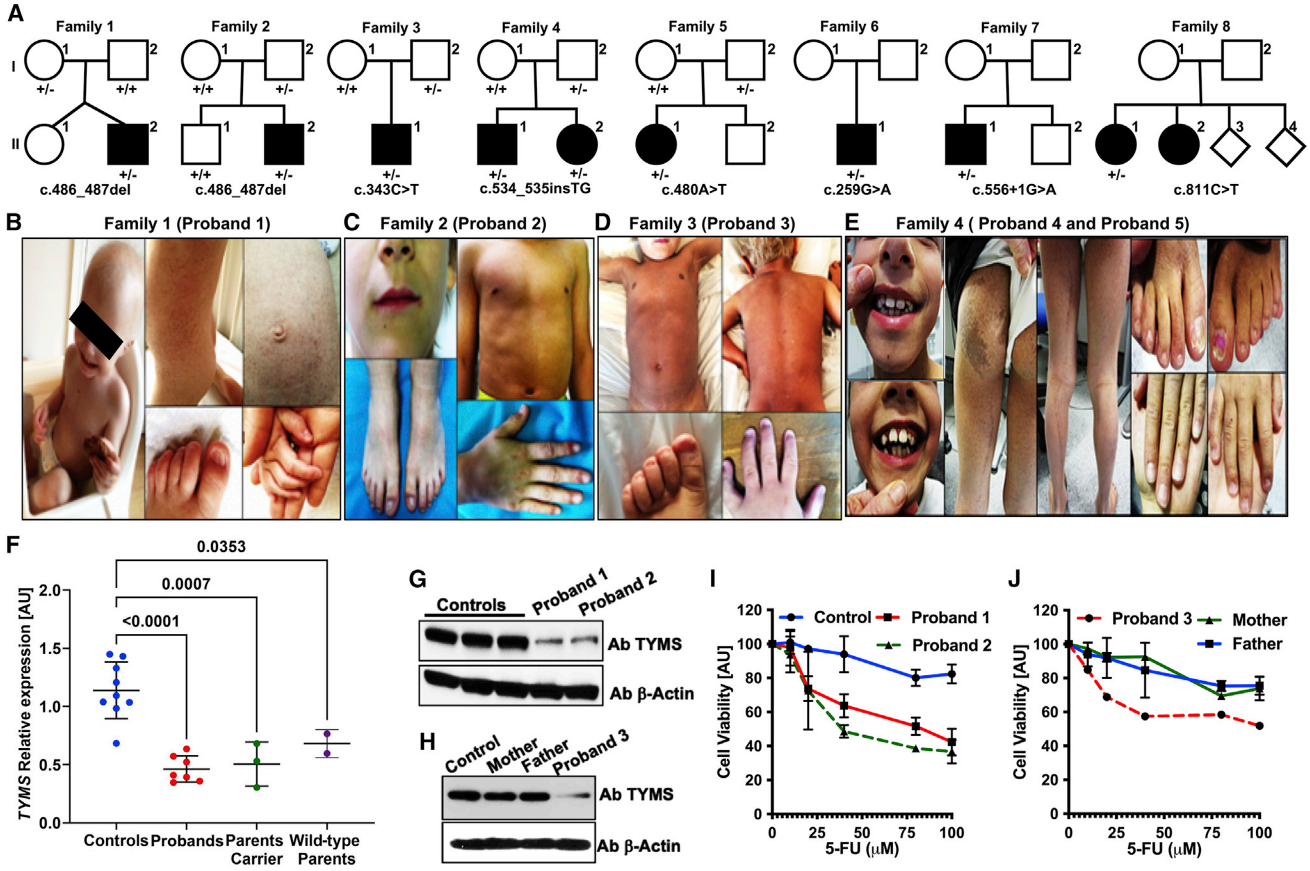

Figure 1. TYMS deficiency in families affected by dyskeratosis congenita

(A) Pedigrees of proband families are as shown and indicate the presence of the *TYMS* variant in the heterozygous (+/−) state. Black circles and squares denote affected probands.

(B–E) Photographs of affected probands show some of the clinical features: sparse hair, nail dystrophy, abnormal skin pigmentation, and abnormal dentition.

(F) Lymphoblastoid cell lines from the probands and parents show reduced levels of *TYMS* expression compared to that in controls. All genes are normalized to *MCM6* and *TFRC*. Data represent means ± SD, n = 3, p values determined by one-way ANNOVA. Samples include cell lines from probands (families 1, 2, and 3) and parents (families 1 and 3) and are compared with unrelated controls.

(G and H) Reduced TYMS protein amounts in individual proband samples from three families are compared to those in parents and controls; β-actin is used as a loading control.

(I and J) 5-fluorouracil (5-FU) sensitivity demonstrating increased toxicity in lymphoblastoid cells from the probands compared to parents and a control.

also remarkable was that despite the clinical heterogeneity of the genetically uncharacterized DC probands included in our exome series, the TYMS families had a consistent phenotype, characterized by an early onset of mucocutaneous features (abnormal skin pigmentation and nail dystrophy) that are the predominant clinical features of classic DC (Figures 1B–1E; Table 1). All variants were verified by Sanger sequencing, and they appeared to affect highly conserved amino acid residues in TYMS (Figures S1 and S2).

Segregation analysis of all available additional family members (families 1–5 only) showed that one parent who was asymptomatic for the disease also harbored the same *TYMS* exonic variant as their affected offspring, thereby excluding an autosomal-dominant pattern of disease inheritance. Quantitative RT-PCR analysis revealed a highly significant reduction in *TYMS* expression in the lymphoblastoid cells derived from affected probands of family 1 (II-2), 2 (II-2), and 3 (II-1) when compared to controls (Figure 1F). The *TYMS* expression appears to be similar between the probands and the unaffected parents carrying either the *TYMS* variant ("parent carrier," family 1 I-1; family 3 I-2) or the wild-type variant ("wild-type parent," family 1 I-2; family 3 I-1) (Figure 1F). The reduction of the *TYMS* RNA expression indicates the presence of a second genetic event in the "wild-type parent" cells. However, upon immunoblotting a stark reduction in TYMS protein level is observed in the cells of affected probands when compared to the parent carrier, wild-type parent, and the controls (Figures 1G and 1H). Furthermore, cells from the affected probands displayed significant hypersensitivity when exposed to the known specific inhibitor of TYMS activity, 5-FU, in comparison to controls and both parents (Figures 1I and 1J). These observations indicate the presence of another genetic variant (or variants)

Table 1. Genotype and clinical phenotype of DC probands with a pathogenic *TYMS* variant

Family	1	2	3	4		5	6	7	8	
TYMS exonic variant	c.486_487 delAA	c.486_487 delAA	c.343C>T	c.534_535 insTG	c.534_535 insTG	c.480A>T	c.259G>A	c.556+1G>A	c.811C>T	ND
Protein change	p.Arg163Ser fsTer3	p.Arg163Ser fsTer3	p.Arg115 Ter	p.Met179 Ter	p.Met179 Ter	p.Gln160His	p.Glu87Lys	?	p.Arg271 Ter	ND
CADD score	33	33	43	33	33	15.98	26.3	25.9	37	ND
Features										
Sex	M	M	M	M	F	F	M	M	F	F
Country/ethnic origin	UK	Italy	UK	UK	UK	USA	China	USA	Germany	Germany
Age at sampling (y)	2	2	3	0.8	1	26	3	4	27	25
Abnormalities in skin pigmentation	Y (1 y)	Y (birth)	Y (1 y)	Y (1 y)	Y (1 y)	Y (1 y)	Y (birth)	Y (1 y)	Y (6 y)	Y
Nail dystrophy	Y (1 y)	Y (birth)	Y (1 y)	Y (1 y)	Y	Y (5 y)	Y (2 y)	Y (2 y)	Y (6 y)	Y
Leukoplakia	Y	N	N	N	N	N	Y	N	Y	Y
Hair loss and thin eye lashes	Y	Y	Y	Y	Y	Y	Y	?	Y	Y
Hematological abnormalities	N[a]	N	N[b]	Y[c]	N	N	N[d]	N	N	N
Immune defects	Y[e]	Y[f]	Y[g]	Y[h]	Y[i]	?	N	Y[j]	?	?
Other features	Y[k]	Y[l]	Y[m]	Y[n]	Y[o]	Y[p]	Y[q]	Y[r]	N	N
Telomere length (Flow-FISH centile)	$<1^{st}$	ND	ND	1st–10th	ND	ND	$<1^{st}$	ND	ND	ND

[a]Normal blood count but raised HbF (1.7%).
[b]Normal blood count but raised HbF (1.2%).
[c]Anemia (98 g/L); other blood counts normal.
[d]Normal blood count but raised HbF (2.3%).
[e]Low IgM.
[f]Low IgA and IgG.
[g]Low IgM.
[h]Low IgA.
[i]Low IgA.
[j]Low IgA.
[k]Recurrent infections in first year, intrauterine growth restriction, gastro-oesophageal reflux, failure to thrive.
[l]Reduction of fingerprints.
[m]Recurrent infections in 1st year.
[n]Short stature, recurrent respiratory and gastro-intestinal infections in first year.
[o]Abnormal teeth.
[p]Bilateral ptosis, tooth discolouration, intermediate increased response to mitomycin-C, basal carcinoma on the chest, squamous carcinoma and melanoma on the leg, severe response to topical 5-FU treatment.
[q]Abnormal facies, dysphagia, microcephaly.
[r]Epiphora, small testes; short stature; F = female, M = male; Y = yes; N = no; ? = unknown; nd = not determined. CADD; combined annotation dependent depletion.

that could be inherited in an autosomal-recessive fashion from the wild-type parent and that has an impact on the overall TYMS expression.

Altered nucleotide metabolism, impaired telomerase regulation, and genome instability are consequences of TYMS deficiency

TYMS participates in the *de novo* nucleotide synthesis pathway by catalyzing the reductive methylation of deoxyuridine monophosphate (dUMP) to form deoxythymidine monophosphate (dTMP; Figure 2A).[29] Analysis of cellular dNTP pool by mass spectrometry revealed an increase in the dUMP nucleotide pools accompanied by a decrease in the dTMP pool as a result of TYMS deficiency affecting the *de novo* pathway for nucleotide synthesis in proband 1's cells (Figures 2B and 2C). We also noticed a substantial increase in the dTTP pool in the proband cells, indicating the activation of the salvage pathway (Figure 2D). Furthermore, a stark increase in thymidine kinase 1 (TK1) amounts correlates with the observation of an increased cellular dTTP pool, whereas a reduction in other key proteins that are involved in initial steps of *de novo* dTMP synthesis is observed in cell lysates of the affected probands (Figure 2E). It has been reported that either the depletion of *de novo* dTMP synthesis or the increase in the cellular dTTP pool, as observed in our affected proband cells, causes genome instability.[30,31]

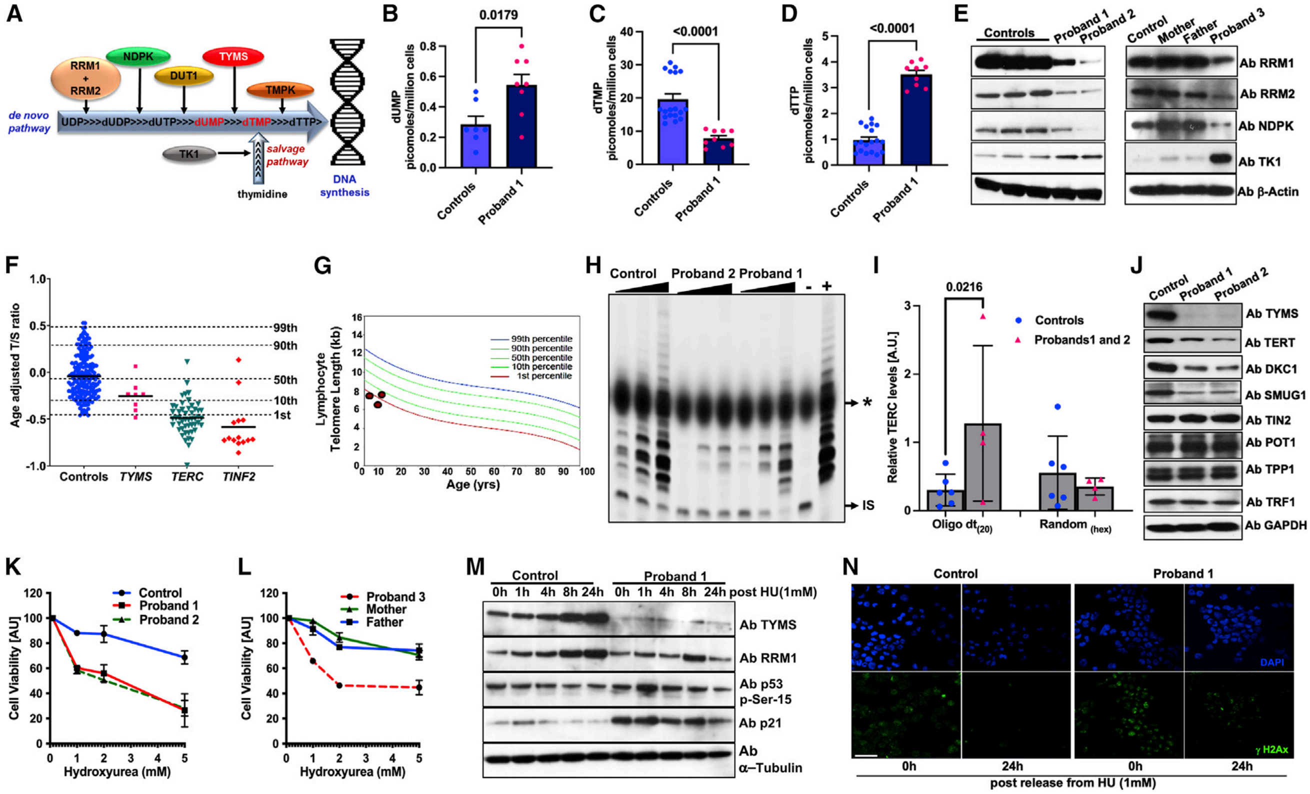

Figure 2. TYMS deficiency impacts nucleotide metabolism, telomere maintenance, and genome instability in probands' cells

(A) Schematic diagram showing the proteins involved in different stages (indicated by arrows) of *de novo* and salvage pathways for dTTP synthesis. Abbreviations are as follows: RRM, ribonucleotide reductase catalytic subunits M1 and M2; NDPK, nucleoside diphosphate kinase; TYMS, thymidylate synthase; and TK1, thymidine kinase 1.

(B–D) The effect of TYMS deficiency on dNTP pools. Cells from both probands and the control were harvested for analysis of dUMP, dTMP, and dTTP pools. The scattered dot plot represents 1 million cells per dot in each set.

(E) Immunoblotting for key proteins in cell lysates of probands compared with unrelated controls. β-actin is used as a loading control.

(F) Relative telomere lengths of probands are reduced in comparison with those of controls. Age-adjusted T/S ratios analyzed by the MMqPCR method show that probands with TYMS variants have shorter telomere lengths. T/S ratios from probands with either *TERC* or *TINF2* variants are shown for comparison.

(G) Telomere length measurement by flow-FISH in probands from families 1, 4, and 6.

(H) Relative levels of telomerase activity in probands and age-matched control cells at passage 2 were determined by TRAP assay. "IS" indicates internal standard, and * refers to the position of loading dye across the lanes.

(I) Oligo-dT$_{(20)}$-primed mature *TERC* RNA transcripts are distinguished from random hexamer priming of cDNA acquired from RNA samples from lymphoblastoid cell lines of probands (box represents mean and whiskers represent standard deviation).

(J) Immunoblots showing levels of DNA-repair protein at steady state in cells from affected probands and controls. GAPDH is used to determine the loading control.

(K and L) Cell viability in cells from probands, parents, and a control in the presence of hydroxyurea.

(M) Immunoblots showing protein level after hydroxyurea treatment. α-tubulin is used as a loading control.

(N) A representative image of γH2AX staining in control and index proband cells 24 h after release from hydroxyurea (HU) treatment. Images show DAPI-stained nuclei in blue and γH2AX in green. The scale bar represents 50 μm.

Telomere length measurement by qPCR and the flow-FISH method revealed short telomere lengths between the first and tenth centiles (Figures 2F and 2G) in the probands. We also observed that probands' cells grew at a slower rate (Figure S3) and demonstrated reduced levels of telomerase activity (Figure 2H), and this decrease was accompanied by an increase in amounts of oligoadenylated immature *TERC* (Figure 2I). The amounts of DKC1, TERT, and SMUG1, which regulate *TERC* stability, also appear to be reduced (Figure 2J), whereas the amount of shelterin protein components (TIN2, TPP1, TRF1 and POT1; Figure 2J), which protect telomeres, remained unchanged, indicating that defects in telomere length maintenance are a consequence of impaired telomerase regulation in these TYMS deficient probands as a result of altered nucleotide metabolism.

Furthermore, an increase in the phosphorylation levels of proteins ATM, CHK1, CHK2, p53, p21 in the DNA-damage-response pathway (Figure S4) and hypersensitivity to ribonucleotide reductase inhibitors such as hydroxyurea (Figures 2K–2L) were observed in the cells of TYMS-deficient probands. Time-course release after exposure to hydroxyurea did not sufficiently restore TYMS or RRM1 protein expression and led to further increase of p53

phosphorylation, p21 (Figure 2M) and γ-H2AX (Figure 2N). Collectively, these results suggest that TYMS deficiency alters the cellular dNTP pools and thereby elevates the DNA-damage response and impairs telomerase regulation; both of these effects are considered to be cellular hallmarks of genetically characterized DC-affected probands.[32,33]

TYMS deficiency occurs via *ENOSF1*-mediated RNA silencing

The severe TYMS deficiency and the hypersensitivity to both 5-FU and hydroxyurea observed in TYMS-deficient proband cells is clearly distinct from cellular characteristics in the parent carrier and the wild-type parent (Figure 1I–1J and 2K–2L). Moreover, TYMS expression appeared to fluctuate over passages at low levels in the proband cells when compared to control cells (Figure S5A). In-cell RNA cross-linking and affinity capture of translating polysomes via HSP70 antibody revealed reduced binding of *TYMS* mRNA to actively translating ribosomes in cells from the affected proband (Figures S5B and S5C). These results clearly demonstrate that reduced TYMS translation is occurring by post-transcriptional inhibition of *TYMS* mRNA binding to translating polysomes in affected proband cells.

ENOSF1 (enolase super family 1 [MIM: 607427]) has been shown to modify TYMS expression at the RNA level by acting as an antisense molecule to *TYMS*.[34,35] *ENOSF1* partially overlaps *TYMS* on chromosome 18 and is transcribed in the opposite direction to *TYMS*. 5-FU is a common treatment in cancer-affected individuals, and many reports demonstrate a pharmacogenetic link between several SNPs in the *TYMS-ENOSF1* locus[36] and the development of severe hand-foot syndrome.[37] These SNPs have reported associations with 5-FU toxicity that results in either the downregulation of *TYMS* or the upregulation of *ENOSF1* expression, or both. On the basis of the observation of 5-FU hypersensitivity in cells from the probands (Figures 1I and 1J), we sequenced this genomic region (chr18: 623000–716000) to screen for any additional germline variants as well as SNPs associated with 5-FU sensitivity.[38–40] We observed three additional rare intronic *ENOSF1* variants that might affect *ENOSF1* expression and one *TYMSOS* (*TYMS* opposite strand) variant that were all inherited from the wild-type parent, (Figures 3A and 3B, Table S3). Although no report to date has described the effect of *TYMSOS* in modifying TYMS expression, the *ENOSF1* transcript has been shown to act as antisense RNA and inhibit TYMS expression. Furthermore, we also identified intronic variants in *ENOSF1* and *TYMS* in the affected probands of family 6 and family 8, respectively, where no parental DNA was unavailable (Table S3). It is noteworthy that a common haplotype is shared by all the probands in families 1–5 and that it is inherited from the parent who does not have the disease-specific *TYMS* variant. This *"C-A-ins"* haplotype (rs699517-rs2790-rs151264360) is associated with both reduced *TYMS* and increased *ENOSF1* expression as well as with severe hand-foot syndrome.[37] This observation of a defined background haplotype in combination with a loss-of-function variant causing severe TYMS deficiency has also been reported in an artificial loss of heterozygosity for the *TYMS* allele in a cancer-cell-line model.[41] This might help to explain the resultant cell phenotype hypersensitivity towards 5-FU in our index probands when compared to the parent carrier (Figure 1J). One of the probands (family 5) who carried *TYMS* exonic missense variant (c.480A>T [p.Gln160His]) has shown a severe adverse response to topical 5-FU when undergoing treatment for her squamous carcinoma and melanoma in her leg (Table 1).

We further evaluated *ENOSF1* RNA expression in cells from the probands and their unaffected heterozygous parents along with controls. This revealed a marked increase in the ratio of *ENOSF1* to *TYMS* expression in proband cells when compared to cells from parents and controls (Figure 3C). This increase in *ENOSF1* expression also appears to be high over passages in the proband cells when compared to control cells (Figure S5D). Lentiviral transduction of GFP-tagged *TYMS* cDNA revealed a clear rescue of endogenous TYMS expression, which ameliorated the *ENOSF1* antisense effect by outcompeting with ectopically expressed *TYMS* RNA in the proband cells (Figures 4A and 4B). This rescue of endogenous TYMS protein appears to be dose dependent and consistent between two other independent transduction experiments in the proband cells (Figures S6A and S6B). We mapped the RNA-RNA interaction region with two different tools, IntaRNA[19] and RactIP,[20] and both identified a putative interaction region spanning nucleotides 564–713 in *TYMS* and 4834–4983 in *ENOSF1,* respectively (Figures S6C and S6D). *RNA* secondary-structure prediction by RNAfold[42] revealed similar folding conformations between *TYMS* and *ENOSF1* RNA molecules and accurate base pairing as determined by IntaRNA and RactIP (Figure 4C). Furthermore, silencing of *ENOSF1* by RNA interference revealed a clear rescue of endogenous TYMS at both the RNA and the protein level in the proband cells (Figure 4D and 4E). This increase of endogenous *TYMS* expression in the cells of the probands in either fashion further revealed a mild or partial but not complete rescue of cell sensitivity to 5-FU (Figure 4F). Thus, our results indicate a digenic epistatic relationship between *TYMS* and *ENOSF1* alleles, where a combination of linked SNPs together with additional rare variants results in increased expression of *ENOSF1* relative to *TYMS.* This leads to post-transcriptional inhibition of TYMS translation by *ENOSF1-TYMS* RNA-RNA interaction, causing severe TYMS deficiency and features of DC in these affected individuals.

Discussion

In this study we identified a cohort of pediatric DC individuals who have a homogeneous mucocutaneous phenotype

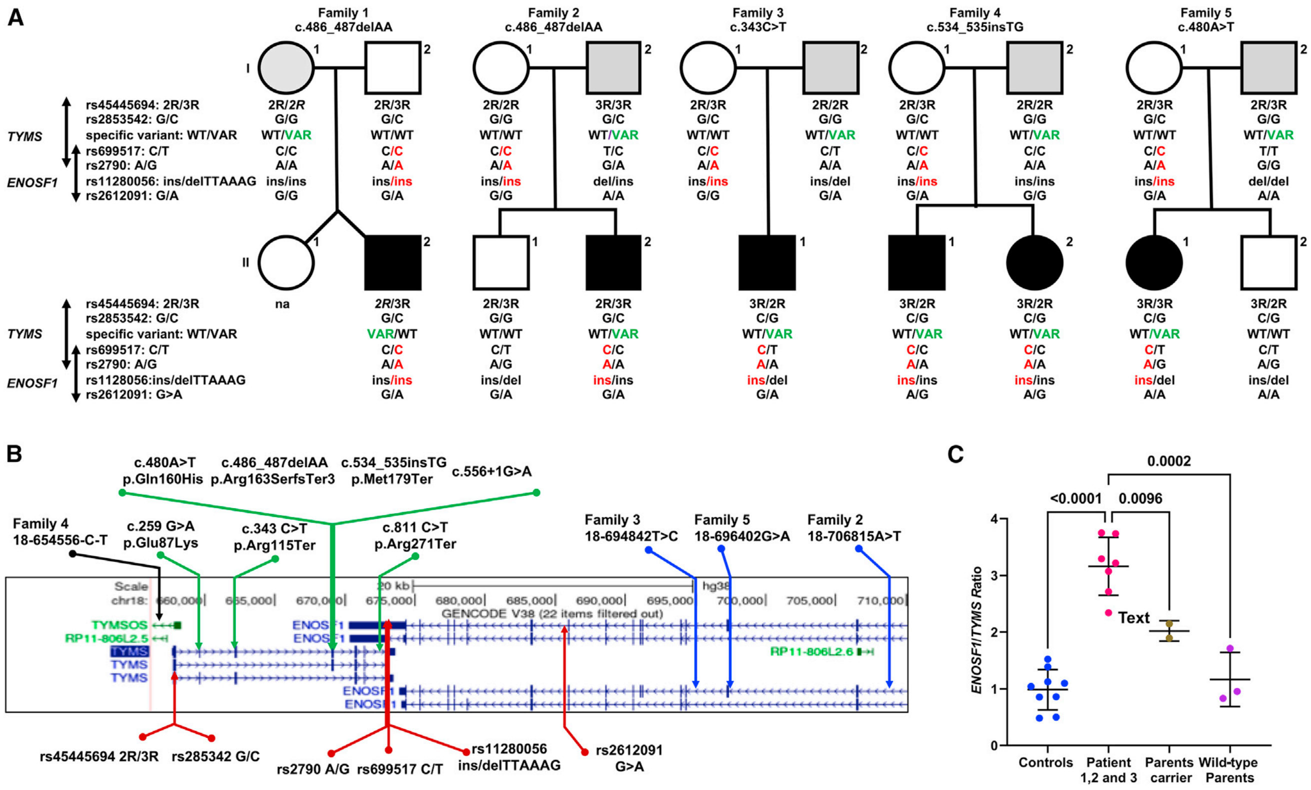

Figure 3. Haplotype analysis and influence of *ENOSF1* variants on *TYMS* expression

(A) Inheritance of common polymorphisms and the variant of interest in the *TYMS-ENOSF1* locus in families I–V. The "*C-A-ins*" haplotype in red highlights the common inherited allele from the wild-type parent. The relative position of the *TYMS*-specific allele is highlighted in green. na indicates that a sample was not available. A black-filled symbol indicates an affected individual; an open symbol indicates an unaffected individual; and a gray-filled symbol indicates an asymptomatic carrier of the *TYMS* exonic variant. The unique 28 bp polymorphic 5′-UTR tandem-repeat sequence that is known as the *TYMS* enhancer region (TSER; rs45445694) and the 6 bp deletion in the 3′ UTR (rs151264360) are shown. The TSER with three polymorphic repeats (3R) has greater *TYMS* expression levels when compared than the two-repeat sequence (2R), and this is further modulated by the presence of SNP G or C (rs2853542) within the 2R when 3R is present.

(B) *TYMS-ENOSF1* genomic locus depicting polymorphisms (red arrows) and intronic variants identified in *ENOSF1* alleles (blue arrows) and the *TYMSOS* allele (black arrow) in individuals for whom parental samples were available. Exonic *TYMS* variants (green arrows) are from all probands in this study. An asterisk indicates a recurrent variant.

(C) *ENOSF1/TYMS* transcript ratio in control and proband cells as well as an unaffected heterozygote parent *TYMS* carrier as analyzed by qPCR.

and harbour previously unreported LOF, missense, and somewhat rare germline variants in the *TYMS* locus that overlaps with *ENOSF1* (Figure 1 and Figure 3). On the basis of the stark TYMS deficiency we observed in the cells of affected probands, we propose that a combined digenic effect of *TYMS* and *ENOSF1* variants modifies *TYMS* expression in triggering disease features in these affected individuals. The parents who are carriers of either of these variants (i.e., affecting only the maternal or paternal *TYMS-ENOSF1* locus) remain asymptomatic (Table S3). These complex germline variants at the *TYMS-ENOSF1* locus introduce an epistatic effect of *ENOSF1* in misbalancing the *TYMS* allele. This epistatic molecular event represents a unique pathogenetic mechanism and provides an important step towards the genetic and molecular understanding of DC.

Previously, a genetic linkage study on a large consanguineous Pakistani family, where affected members manifested clinical features of syndromic ectodermal dysplasia [ECTD (MIM: 616029)], has revealed a linkage candidate region on chromosome 18p11.32-p11.3, which includes the *TYMS-ENOSF1* locus.[43] However, further sequencing did not find that this region harbors any homozygous or biallelic variants segregating with the ECTD phenotype observed in this family. Because somatic features (hair, nails, teeth, and skin pigmentation) of ECTD individuals often overlap with DC,[16] it is tempting to speculate that a combination of variants in this linkage region modify the status of *TYMS* and *ENOSF1* expression and cause ECTD- or DC-like features in this family.

The relative increase in the ratio of *ENOSF1* to *TYMS* transcripts in three independent cell lines obtained from our affected probands (Figure 3C) suggests that high *ENOSF1* expression reduces *TYMS* translation by inhibiting *TYMS* binding to translating ribosomes via an antisense mechanism (Figures S5B and S5C). This result implies that it is the relative levels of these transcripts (high *ENOSF1/TYMS* ratio), which in turn determines the overall

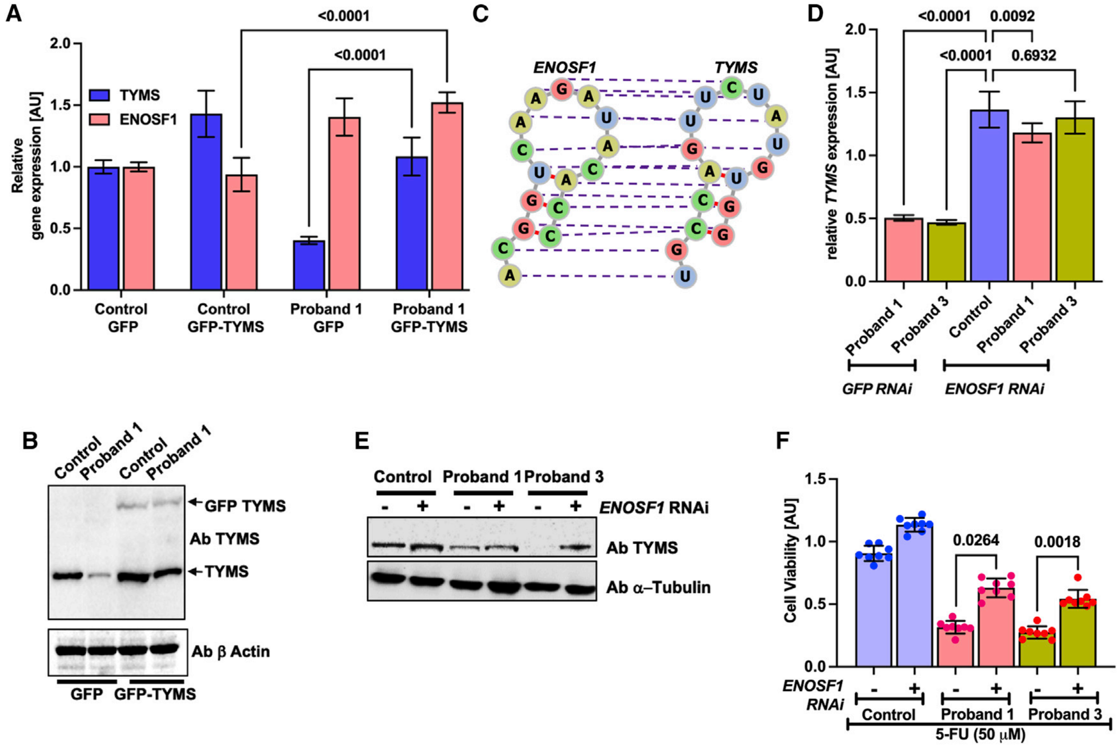

Figure 4. Post-transcriptional epistatic silencing of *TYMS* by elevated *ENOSF1* in cells of the affected probands
(A) RNA expression of *TYMS* and *ENOSF1* after rescue by a GFP-TYMS lentiviral particle. Expression is relative to the control-GFP in each proband.
(B) Immunoblotting of TYMS protein in control and proband cells transduced with lentivirus particles encoding GFP alone and GFP-tagged *TYMS* cDNA.
(C) The RNA secondary structures of both *TYMS* and *ENOSF1* in this RactIP predicted region is modelled with the RNAfold webserver under default parameters. The purple-colored dashed lines indicate base pairing of RNA residues between *TYMS* and *ENOSF1*.
(D) RNA expression of *TYMS* and *ENOSF1* after transduction with lentiviral particles encoding *ENOSF1* shRNA. Expression is relative to the control-GFP shRNA in each proband.
(E) Immunoblotting of TYMS protein in control and proband cells transduced with lentivirus particles encoding *ENOSF1* shRNA.
(F) Cellular sensitivity to 5-flurouracil (5-FU) in control and proband cells after transduction of lentivirus particles encoding *ENOSF1 RNAi*. For (A), (D), and (F), each experiment was performed in duplicate and analyzed in triplicate.

TYMS accumulation and the resulting DC phenotype. Specifically, the exonic *TYMS* variant on one parental chromosome leads to the reduction or absence of *TYMS* expression from that allele. Combined with increased expression of *ENOSF1* from the other parental chromosome, this leads to a further reduction from its *cis TYMS* locus, resulting in severe TYMS deficiency as observed in cells from our affected probands (Figures 1G–1H). The altered *ENOSF1/TYMS* ratio could also be the underlying basis of the severe hand-foot syndrome that is observed in a subset of cancer-affected individuals treated with 5-fluorouracil and related cancer drugs.

As a molecular consequence of TYMS deficiency in the cells from probands, alteration in the nucleotide metabolism pathway caused an elevated DNA-damage response and induced the p53/p21 axis, affecting the expression of key proteins (DKC1, SMUG1, and TERT) that are involved in telomerase regulation (Figure 2). These molecular events are considered senescence hallmarks that impair stem cell renewal.[44] In DC and the related inherited bone-marrow failure syndrome Fanconi anaemia, loss of the hematopoietic stem cell niche is accompanied by activation of the p53/p21 axis, inducing a replicative senescent phenotype.[45–47] Furthermore, studies on transgenic mouse models of nucleotide deficiency reported that the fine-tuning of nucleotide metabolism pathways is required for resolution of replication stress and overcoming maturation defects of hematopoietic stem and progenitor cells *in vivo*.[48]

Nucleotide-metabolism disorders represent diverse clinical manifestations, including neurological, immunological, hematological, and renal impairments; adverse reactions to 5-FU therapy; and association with malignancies.[49] Bi-allelic *DPYD, TK2*, and *TYMP* variants that participate in the *de novo* synthesis pathway have been reported in individuals manifesting the neurogastrointestinal and skeletal-muscle aging disorder dihydropyramidine dehydrogenase deficiency (DYD [MIM: 274270]) and mitochondrial DNA depletion syndrome types 1 and 2 (MTDPS1 and 2 [MIM: 609560; MIM: 603041]).[50–52]

Recently, bi-allelic *DUT* (deoxyuridine triphosphatase [MIM: 601266]) variants that hydrolyses dUTP to dUMP

in the *de novo* synthesis pathway were reported in individuals with bone-marrow failure associated with diabetes.[53] In our exomes, we also identified probands who came from two independent families, had bi-allelic *DUT* variants, and presented with severe pancytopenia and mucocutaneous skin features (Figure S7). The imbalance that occurs in dNTP pools as a result of variants in the aforementioned genes has been shown to affect mitochondrial DNA more adversely than nuclear DNA. In light of the previous observations that TYMS subcellular localization to mammalian mitochondria regulates the *de novo* synthesis pathway for faithful mitochondrial DNA replication,[54] it is possible that our reported probands with severe TYMS deficiency have some defects in mitochondrial function.

In summary, our study provides evidence that germline variants at the *TYMS-ENOSF1* locus give rise to severe thymidylate synthase deficiency that disrupts the nucleotide metabolism pathway and that this disruption drives molecular features of genome instability and senescence in a homogenous cohort of DC individuals. These observations highlight the influence of nucleotide-metabolism genes in genome stability and demonstrate that digenic germline variants in both *TYMS* and *ENOSF1* can generate disease features of dyskeratosis congenita.

Data and code availability

The published article includes all genomic variants analyzed during this study. The variant data have been submitted to the ClinVar database under accession numbers SCV002540636 and SCV002540642.

Supplemental information

Supplemental information can be found online at https://doi.org/10.1016/j.ajhg.2022.06.014.

Acknowledgments

We would like to thank all the affected probands' families for their participation in this study. We acknowledge financial support provided by UKRI/MRC (MR/P018440/1) and Blood Cancer UK (14032). We would like to thank Patrick Shannon and Enrico Colombo for sending the samples from affected individuals.

Author contributions

Conceptualization and investigation, H.T., A.W., T.V., and I.D.; methodology, H.T., A.W., J.A., R.B., P.M., M.P., and T.V.; data analysis, H.T., A.W., N.P., A.S., T.V., and I.D.; resources, P.R., A.M.R., M.W., S.C., A.M.E., V.K., V.C., T.V., and I.D.; writing—original draft, HT; writing—review and editing, HT, AW, TV, ID; Supervision and Funding acquisition, TV, ID.

Declaration of interests

The authors declare no competing interest.

Received: April 28, 2022
Accepted: June 23, 2022
Published: August 4, 2022

Web resources

ClinVar, https://www.ncbi.nlm.nih.gov/clinvar/

Genome Aggregation database (gnomAD), https://gnomad.broadinstitute.org

INTARNA prediction, http://rna.informatik.uni-freiburg.de/IntaRNA/

Online Mendelian Inheritance in Man (OMIM), http://www.omim.org

RNA secondary structure prediction, http://rtips.dna.bio.keio.ac.jp/ractip/

References

1. Dokal, I., Tummala, H., and Vulliamy, T.J. (2022). Inherited bone marrow failure in the pediatric patient. Blood. blood. 2020006481. https://doi.org/10.1182/blood.2020006481.
2. Zinsser, F. (1910). Atrophia Cutis Reticularis cum Pigmentations, Dystrophia Unguium et Leukoplakis oris (Poikioodermia atrophicans vascularis Jacobi.). Ikonographia Dermatologica *5*, 219–223.
3. Tummala, H., Walne, A., Collopy, L., Cardoso, S., de la Fuente, J., Lawson, S., Powell, J., Cooper, N., Foster, A., Mohammed, S., et al. (2015). Poly(A)-specific ribonuclease deficiency impacts telomere biology and causes dyskeratosis congenita. J. Clin. Invest. *125*, 2151–2160. https://doi.org/10.1172/jci78963.
4. Gable, D.L., Gaysinskaya, V., Atik, C.C., Talbot, C.C., Jr., Kang, B., Stanley, S.E., Pugh, E.W., Amat-Codina, N., Schenk, K.M., Arcasoy, M.O., et al. (2019). ZCCHC8, the nuclear exosome targeting component, is mutated in familial pulmonary fibrosis and is required for telomerase RNA maturation. Genes Dev. *33*, 1381–1396. https://doi.org/10.1101/gad.326785.119.
5. Stanley, S.E., Gable, D.L., Wagner, C.L., Carlile, T.M., Hanumanthu, V.S., Podlevsky, J.D., Khalil, S.E., DeZern, A.E., Rojas-Duran, M.F., Applegate, C.D., et al. (2016). Loss-of-function mutations in the RNA biogenesis factor NAF1 predispose to pulmonary fibrosis-emphysema. Sci. Transl. Med. *8*, 351ra107. https://doi.org/10.1126/scitranslmed.aaf7837.
6. O'Connor, M.S., Safari, A., Xin, H., Liu, D., and Songyang, Z. (2006). A critical role for TPP1 and TIN2 interaction in high-order telomeric complex assembly. Proc. Natl. Acad. Sci. USA *103*, 11874–11879. https://doi.org/10.1073/pnas.0605303103.
7. Nandakumar, J., Bell, C.F., Weidenfeld, I., Zaug, A.J., Leinwand, L.A., and Cech, T.R. (2012). The TEL patch of telomere protein TPP1 mediates telomerase recruitment and processivity. Nature *492*, 285–289. https://doi.org/10.1038/nature11648.
8. Kelich, J., Aramburu, T., van der Vis, J.J., Showe, L., Kossenkov, A., van der Smagt, J., Massink, M., Schoemaker, A., Hennekam, E., Veltkamp, M., et al. (2022). Telomere dysfunction implicates POT1 in patients with idiopathic pulmonary fibrosis. J. Exp. Med. *219*, e20211681. https://doi.org/10.1084/jem.20211681.

9. Venteicher, A.S., Abreu, E.B., Meng, Z., McCann, K.E., Terns, R.M., Veenstra, T.D., Terns, M.P., and Artandi, S.E. (2009). A human telomerase holoenzyme protein required for Cajal body localization and telomere synthesis. Science *323*, 644–648. https://doi.org/10.1126/science.1165357.
10. Wang, F., Stewart, J.A., Kasbek, C., Zhao, Y., Wright, W.E., and Price, C.M. (2012). Human CST has independent functions during telomere duplex replication and C-strand fill-in. Cell Rep. *2*, 1096–1103. https://doi.org/10.1016/j.celrep.2012.10.007.
11. Simon, A.J., Lev, A., Zhang, Y., Weiss, B., Rylova, A., Eyal, E., Kol, N., Barel, O., Cesarkas, K., Soudack, M., et al. (2016). Mutations in STN1 cause Coats plus syndrome and are associated with genomic and telomere defects. J. Exp. Med. *213*, 1429–1440. https://doi.org/10.1084/jem.20151618.
12. Vannier, J.B., Pavicic-Kaltenbrunner, V., Petalcorin, M.I., Ding, H., and Boulton, S.J. (2012). RTEL1 dismantles T loops and counteracts telomeric G4-DNA to maintain telomere integrity. Cell *149*, 795–806. https://doi.org/10.1016/j.cell.2012.03.030.
13. Toufektchan, E., Lejour, V., Durand, R., Giri, N., Draskovic, I., Bardot, B., Laplante, P., Jaber, S., Alter, B.P., Londono-Vallejo, J.A., et al. (2020). Germline mutation of MDM4, a major p53 regulator, in a familial syndrome of defective telomere maintenance. Sci. Adv. *6*, eaay3511. https://doi.org/10.1126/sciadv.aay3511.
14. Sharma, R., Sahoo, S.S., Honda, M., Granger, S.L., Goodings, C., Sanchez, L., Künstner, A., Busch, H., Beier, F., Pruett-Miller, S.M., et al. (2022). Gain-of-function mutations in RPA1 cause a syndrome with short telomeres and somatic genetic rescue. Blood *139*, 1039–1051. https://doi.org/10.1182/blood.2021011980.
15. Nachmani, D., Bothmer, A.H., Grisendi, S., Mele, A., Bothmer, D., Lee, J.D., Monteleone, E., Cheng, K., Zhang, Y., Bester, A.C., et al. (2019). Germline NPM1 mutations lead to altered rRNA 2′-O-methylation and cause dyskeratosis congenita. Nat. Genet. *51*, 1518–1529. https://doi.org/10.1038/s41588-019-0502-z.
16. Walne, A.J., Collopy, L., Cardoso, S., Ellison, A., Plagnol, V., Albayrak, C., Albayrak, D., Kilic, S.S., Pat roglu, T., Akar, H., et al. (2016). Marked overlap of four genetic syndromes with dyskeratosis congenita confounds clinical diagnosis. Haematologica *101*, 1180–1189. https://doi.org/10.3324/haematol.2016.147769.
17. Hilcenko, C., Simpson, P.J., Finch, A.J., Bowler, F.R., Churcher, M.J., Jin, L., Packman, L.C., Shlien, A., Campbell, P., Kirwan, M., et al. (2013). Aberrant 3′ oligoadenylation of spliceosomal U6 small nuclear RNA in poikiloderma with neutropenia. Blood *121*, 1028–1038. https://doi.org/10.1182/blood-2012-10-461491.
18. Pontikos, N., Yu, J., Moghul, I., Withington, L., Blanco-Kelly, F., Vulliamy, T., Wong, T.L.E., Murphy, C., Cipriani, V., Fiorentino, A., et al. (2017). Phenopolis: an open platform for harmonization and analysis of genetic and phenotypic data. Bioinformatics *33*, 2421–2423. https://doi.org/10.1093/bioinformatics/btx147.
19. Mann, M., Wright, P.R., and Backofen, R. (2017). IntaRNA 2.0: enhanced and customizable prediction of RNA-RNA interactions. Nucleic Acids Res. *45*, W435–W439. https://doi.org/10.1093/nar/gkx279.
20. Kato, Y., Sato, K., Asai, K., and Akutsu, T. (2012). Rtips: fast and accurate tools for RNA 2D structure prediction using integer programming. Nucleic Acids Res. *40*, W29–W34. https://doi.org/10.1093/nar/gks412.
21. Kudo, K., Xi, Y., Wang, Y., Song, B., Chu, E., Ju, J., Russo, J.J., and Ju, J. (2010). Translational control analysis by translationally active RNA capture/microarray analysis (TrIP-Chip). Nucleic Acids Res. *38*, e104. https://doi.org/10.1093/nar/gkq024.
22. Cawthon, R.M. (2009). Telomere length measurement by a novel monochrome multiplex quantitative PCR method. Nucleic Acids Res. *37*, e21. https://doi.org/10.1093/nar/gkn1027.
23. Baerlocher, G.M., Vulto, I., de Jong, G., and Lansdorp, P.M. (2006). Flow cytometry and FISH to measure the average length of telomeres (flow FISH). Nat. Protoc. *1*, 2365–2376. https://doi.org/10.1038/nprot.2006.263.
24. Chen, P., Liu, Z., Liu, S., Xie, Z., Aimiuwu, J., Pang, J., Klisovic, R., Blum, W., Grever, M.R., Marcucci, G., and Chan, K.K. (2009). A LC-MS/MS method for the analysis of intracellular nucleoside triphosphate levels. Pharm Res. *26*, 1504–1515. https://doi.org/10.1007/s11095-009-9863-9.
25. Codd, V., Wang, Q., Allara, E., Musicha, C., Kaptoge, S., Stoma, S., Jiang, T., Hamby, S.E., Braund, P.S., Bountziouka, V., et al. (2021). Polygenic basis and biomedical consequences of telomere length variation. Nat. Genet. *53*, 1425–1433. https://doi.org/10.1038/s41588-021-00944-6.
26. Li, C., Stoma, S., Lotta, L.A., Warner, S., Albrecht, E., Allione, A., Arp, P.P., Broer, L., Buxton, J.L., Da Silva Couto Alves, A., et al. (2020). Genome-wide association analysis in humans links nucleotide metabolism to leukocyte telomere length. Am. J. Hum. Genet. *106*, 389–404. https://doi.org/10.1016/j.ajhg.2020.02.006.
27. Dorajoo, R., Chang, X., Gurung, R.L., Li, Z., Wang, L., Wang, R., Beckman, K.B., Adams-Haduch, J., Yiamunaa, M., Liu, S., et al. (2019). Loci for human leukocyte telomere length in the Singaporean Chinese population and trans-ethnic genetic studies. Nat. Commun. *10*, 2491. https://doi.org/10.1038/s41467-019-10443-2.
28. Taub, M.A., Conomos, M.P., Keener, R., Iyer, K.R., Weinstock, J.S., Yanek, L.R., Lane, J., Miller-Fleming, T.W., Brody, J.A., Raffield, L.M., et al. (2022). Genetic determinants of telomere length from 109, 122 ancestrally diverse whole-genome sequences in TOPMed. Cell Genomics *2*, 100084. https://doi.org/10.1016/j.xgen.2021.100084.
29. Samsonoff, W.A., Reston, J., McKee, M., O'Connor, B., Galivan, J., Maley, G., and Maley, F. (1997). Intracellular location of thymidylate synthase and its state of phosphorylation. J. Biol. Chem. *272*, 13281–13285. https://doi.org/10.1074/jbc.272.20.13281.
30. Blount, B.C., Mack, M.M., Wehr, C.M., MacGregor, J.T., Hiatt, R.A., Wang, G., Wickramasinghe, S.N., Everson, R.B., and Ames, B.N. (1997). Folate deficiency causes uracil misincorporation into human DNA and chromosome breakage: implications for cancer and neuronal damage. Proc. Natl. Acad. Sci. USA *94*, 3290–3295. https://doi.org/10.1073/pnas.94.7.3290.
31. Ke, P.Y., Kuo, Y.Y., Hu, C.M., and Chang, Z.F. (2005). Control of dTTP pool size by anaphase promoting complex/cyclosome is essential for the maintenance of genetic stability. Genes Dev. *19*, 1920–1933. https://doi.org/10.1101/gad.1322905.
32. Kirwan, M., Beswick, R., Walne, A.J., Hossain, U., Casimir, C., Vulliamy, T., and Dokal, I. (2011). Dyskeratosis congenita and the DNA damage response. Br. J. Haematol. *153*, 634–643. https://doi.org/10.1111/j.1365-2141.2011.08679.x.

33. Pereboeva, L., Westin, E., Patel, T., Flaniken, I., Lamb, L., Klingelhutz, A., and Goldman, F. (2013). DNA damage responses and oxidative stress in dyskeratosis congenita. PLoS One *8*, e76473. https://doi.org/10.1371/journal.pone.0076473.
34. Wu, Q., and Dolnick, B.J. (2003). Detection of thymidylate synthase modulators by a novel screening assay. Mol. Pharmacol. *63*, 167–173. https://doi.org/10.1124/mol.63.1.167.
35. Dolnick, B.J. (1993). Cloning and characterization of a naturally occurring antisense RNA to human thymidylate synthase mRNA. Nucleic Acids Res. *21*, 1747–1752. https://doi.org/10.1093/nar/21.8.1747.
36. Schaerer, D., Froehlich, T.K., Hamzic, S., Offer, S.M., Diasio, R.B., Joerger, M., Amstutz, U., and Largiadèr, C.R. (2020). A novel nomenclature for repeat motifs in the thymidylate synthase enhancer region and its relevance for pharmacogenetic studies. J. Pers. Med. *10*, 181. https://doi.org/10.3390/jpm10040181.
37. Hamzic, S., Kummer, D., Froehlich, T.K., Joerger, M., Aebi, S., Palles, C., Thomlinson, I., Meulendijks, D., Schellens, J.H.M., García-González, X., et al. (2020). Evaluating the role of ENOSF1 and TYMS variants as predictors in fluoropyrimidine-related toxicities: an IPD meta-analysis. Pharmacol. Res. *152*, 104594. https://doi.org/10.1016/j.phrs.2019.104594.
38. Horie, N., Aiba, H., Oguro, K., Hojo, H., and Takeishi, K. (1995). Functional analysis and DNA polymorphism of the tandemly repeated sequences in the 5′-terminal regulatory region of the human gene for thymidylate synthase. Cell Struct. Funct. *20*, 191–197. https://doi.org/10.1247/csf.20.191.
39. Kawakami, K., Salonga, D., Park, J.M., Danenberg, K.D., Uetake, H., Brabender, J., Omura, K., Watanabe, G., and Danenberg, P.V. (2001). Different lengths of a polymorphic repeat sequence in the thymidylate synthase gene affect translational efficiency but not its gene expression. Clin. Cancer Res. *7*, 4096–4101.
40. Nief, N., Morvan, V.L., and Robert, J. (2007). Involvement of gene polymorphisms of thymidylate synthase in gene expression, protein activity and anticancer drug cytotoxicity using the NCI-60 panel. Eur. J. Cancer *43*, 955–962. https://doi.org/10.1016/j.ejca.2006.12.012.
41. Brody, J.R., Hucl, T., Gallmeier, E., Winter, J.M., Kern, S.E., and Murphy, K.M. (2006). Genomic copy number changes affecting the thymidylate synthase (TYMS) gene in cancer: a model for patient classification to aid fluoropyrimidine therapy. Cancer Res. *66*, 9369–9373. https://doi.org/10.1158/0008-5472.can-06-2165.
42. Gruber, A.R., Lorenz, R., Bernhart, S.H., Neubock, R., and Hofacker, I.L. (2008). The Vienna RNA websuite. Nucleic Acids Res. *36*, W70–W74. https://doi.org/10.1093/nar/gkn188.
43. Habib, R., Ansar, M., Mattheisen, M., Shahid, M., Ali, G., Ahmad, W., and Betz, R.C. (2015). A novel locus for ectodermal dysplasia of hair, nail and skin pigmentation anomalies maps to chromosome 18p11.32-p11.31. PLoS One *10*, e0129811. https://doi.org/10.1371/journal.pone.0129811.
44. Gorgoulis, V., Adams, P.D., Alimonti, A., Bennett, D.C., Bischof, O., Bishop, C., Campisi, J., Collado, M., Evangelou, K., Ferbeyre, G., et al. (2019). Cellular senescence: defining a path forward. Cell *179*, 813–827. https://doi.org/10.1016/j.cell.2019.10.005.
45. Batista, L.F.Z., Pech, M.F., Zhong, F.L., Nguyen, H.N., Xie, K.T., Zaug, A.J., Crary, S.M., Choi, J., Sebastiano, V., Cherry, A., et al. (2011). Telomere shortening and loss of self-renewal in dyskeratosis congenita induced pluripotent stem cells. Nature *474*, 399–402. https://doi.org/10.1038/nature10084.
46. Westin, E.R., Aykin-Burns, N., Buckingham, E.M., Spitz, D.R., Goldman, F.D., and Klingelhutz, A.J. (2011). The p53/p21(WAF/CIP) pathway mediates oxidative stress and senescence in dyskeratosis congenita cells with telomerase insufficiency. Antioxid. Redox Signal *14*, 985–997. https://doi.org/10.1089/ars.2010.3444.
47. Ceccaldi, R., Parmar, K., Mouly, E., Delord, M., Kim, J.M., Regairaz, M., Pla, M., Vasquez, N., Zhang, Q.S., Pondarre, C., et al. (2012). Bone marrow failure in Fanconi anemia is triggered by an exacerbated p53/p21 DNA damage response that impairs hematopoietic stem and progenitor cells. Cell Stem Cell *11*, 36–49. https://doi.org/10.1016/j.stem.2012.05.013.
48. Austin, W.R., Armijo, A.L., Campbell, D.O., Singh, A.S., Hsieh, T., Nathanson, D., Herschman, H.R., Phelps, M.E., Witte, O.N., Czernin, J., and Radu, C.G. (2012). Nucleoside salvage pathway kinases regulate hematopoiesis by linking nucleotide metabolism with replication stress. J. Exp. Med. *209*, 2215–2228. https://doi.org/10.1084/jem.20121061.
49. Balasubramaniam, S., Duley, J.A., and Christodoulou, J. (2014). Inborn errors of pyrimidine metabolism: clinical update and therapy. J. Inherit. Metab. Dis. *37*, 687–698. https://doi.org/10.1007/s10545-014-9742-3.
50. Vreken, P., Van Kuilenburg, A.B.P., Meinsma, R., and van Gennip, A.H. (1997). Identification of novel point mutations in the dihydropyrimidine dehydrogenase gene. J. Inherit. Metab. Dis. *20*, 335–338. https://doi.org/10.1023/a:1005357307122.
51. Nishino, I., Spinazzola, A., and Hirano, M. (1999). Thymidine phosphorylase gene mutations in MNGIE, a human mitochondrial disorder. Science *283*, 689–692. https://doi.org/10.1126/science.283.5402.689.
52. Saada, A., Shaag, A., Mandel, H., Nevo, Y., Eriksson, S., and Elpeleg, O. (2001). Mutant mitochondrial thymidine kinase in mitochondrial DNA depletion myopathy. Nat. Genet. *29*, 342–344. https://doi.org/10.1038/ng751.
53. Dos Santos, R.S., Daures, M., Philippi, A., Romero, S., Marselli, L., Marchetti, P., Senée, V., Bacq, D., Besse, C., Baz, B., et al. (2017). dUTPase (DUT) is mutated in a novel monogenic syndrome with diabetes and bone marrow failure. Diabetes *66*, 1086–1096. https://doi.org/10.2337/db16-0839.
54. Anderson, D.D., Quintero, C.M., and Stover, P.J. (2011). Identification of a de novo thymidylate biosynthesis pathway in mammalian mitochondria. Proc. Natl. Acad. Sci. USA *108*, 15163–15168. https://doi.org/10.1073/pnas.1103623108.

The American Journal of Human Genetics, Volume *109*

Supplemental information

Germline thymidylate synthase deficiency impacts nucleotide metabolism and causes dyskeratosis congenita

Hemanth Tummala, Amanda Walne, Roberto Buccafusca, Jenna Alnajar, Anita Szabo, Peter Robinson, Allyn McConkie-Rosell, Meredith Wilson, Suzanne Crowley, Veronica Kinsler, Anna-Maria Ewins, Pradeepa M. Madapura, Manthan Patel, Nikolas Pontikos, Veryan Codd, Tom Vulliamy, and Inderjeet Dokal

Supplementary Information

Table S1: Antibodies used in the study

Antibody	Company	Catalogue number	Dilution
TYMS	Abcam	ab108995	WB 1:2000
TYMK (TK1)	Cell signalling	8960S	WB 1:1000
RRM1	antibodies-online GmbH	ABIN2150979	WB 1:1000
RRM2	Insight Biotechnology	GTX124441-S	WB 1:1000
HSP70	Enzo life sciences	ADI-SPA-819-D	CoIP 1:500
HSP70	Insight Biotechnology	SAB-40416-1	CoIP 1:500
NDPK	antibodies-online GmbH	ABIN5547672	WB 1:1000
β Actin	Abcam	ab8227	WB 1:3000
α tubulin	Abcam	ab7291	WB 1:3000
GAPDH	Abcam	ab8245	WB 1:3000
ATM	Abcam	ab78	WB 1:1000
Phospho-ATM Serine 1981	Abcam	ab208775	WB 1:1000
CHK1	Abcam	ab79758	WB 1:1000
Phospho-CHK1 Serine 345	Abcam	ab58567	WB 1:1000
CHK2	Abcam	ab109413	WB 1:1000
Phospho-CHK2 Threonine 68	Abcam	ab32418	WB 1:1000
TP53	Cell signalling	48818S	WB 1:1000
Phospho p53 Serine 15	Cell signalling	9284T	WB 1:1000
P21	Abcam	AB109520	WB 1:500
DKC1	Abcam	EPR10398	WB 1:1000
TERT	Abcam	ab32020	WB 1:1000
SMUG	Abcam	ab192240	WB 1:1000
TIN2	Abcam	ab197894	WB 1:1000
POT1	Enzo life sciences	PSC-15-787-R050	WB 1:1000
TPP1	antibodies-online GmbH	ABIN5074829	WB 1:1000
TRF1	Insight Biotechnology	TA309899	WB 1:1000
H2AX serine 139	Cell signalling	9718S	IF 1:300

Source of antibody procurement and relevant dilutions used in experiments. WB refers to western blot and ICC refers to immuno cyto chemistry.

Table S2: Bone marrow failure gene panel

ACD	G6PC3	RPL5
ADA2	GATA1	RPL9
ANKRD26	GATA2	RPS10
BRCA1	GFI1	RPS17
BRCA2	GRHL2	RPS19
BRIP1	HAX1	RPS24
C15orf41	HOXA11	RPS26
CDAN1	JAGN1	RPS27
CEBPA	KIF23	RPS28
CSF3R	KLF1	RPS29
CTC1	LIG4	RPS7
CXCR4	LPIN2	RTEL1
CYCS	MAD2L2	RUNX1
DCLRE1B	MECOM	SAMD9
DDX41	MPL	SAMD9L
DKC1	MYSM1	SBDS
DNAJC21	NAF1	SEC23B
DNAJC3	NHP2	SHQ1
DUT	NOP10	SLX4
EFL1	NPM1	SP1
ELANE	PALB2	SRP54
ERBB3	PARN	SRP72
ERCC4	POLA1	STN1
ERCC6L2	POT1	TAZ
ETV6	RAD51	TERC
FANCA	RAD51C	TERT
FANCB	RBM8A	THPO
FANCC	RECQL4	TINF2
FANCD2	RFWD3	TP53
FANCE	RMRP	TYMS
FANCF	RPL11	UBE2T
FANCG	RPL15	USB1
FANCI	RPL18	VPS45
FANCL	RPL26	WAS
FANCM	RPL27	WRAP53
FYB1	RPL31	XRCC2
G6PC	RPL35A	ZCCHC8

Table S3: Variants in *TYMS*- *ENOSF1* locus

Family	Individual	*TYMS* Variant	Additional variant (NC_000018.10: g.623000-716000)	gnomAD MAF	Location
1	father	.	.	.	.
1	Index/proband (M)	c.485_487delAA: pR163SfsTer3	.	.	.
1	mother	c.485_487delAA: pR163SfsTer3	.	.	.
2	father	c.485_487delAA: pR163SfsTer3	.	.	.
2	Index/proband (M)	c.485_487delAA: pR163SfsTer3	NC_000018.10:g.706815A>T (LCR)	NR	*ENOSF1*, intron 1
2	mother	.	NC_000018.10:g.706815A>T (LCR)	NR	*ENOSF1*, intron 1
2	U/A brother	.	.	.	.
3	father	c.343C>T: p.R115X	.	.	.
3	Index/proband (M)	c.343C>T: p.R115X	NC_000018.10:g.694842T>C	0.0009	*ENOSF1*, intron 3
3	mother	.	NC_000018.10:g.694842T>C	0.0009	*ENOSF1*, intron 3
4	affected sister	c.534_535insTG: p.M179X	NC_000018.10:g.694842T>C	0.00026	TYMSOS, intron 1
4	father	c.534_535insTG: p.M179X	.	.	.
4	Index/proband (M)	c.534_535insTG: p.M179X	NC_000018.10:g.654556C>T	0.00026	*TYMSOS*, intron 1
4	mother	.	NC_000018.10:g.654556C>T	0.00026	*TYMSOS*, intron 1
5	father	c.A480T: p.Q160H	.	.	.
5	Index/proband (F)	c.A480T: p.Q160H	NC_000018.10:g.696402C>T	0.0013	*ENOSF1*, intron 3
5	mother	.	NC_000018.10:g.696402C>T	0.0013	*ENOSF1*, intron 3
5	U/A brother	.	.	.	.
6	Index/proband (M)	c.G259A: p.E87K	NC_000018.10:g.694158C>T	NR	*ENOSF1*, intron 4
7	Index/Proband (M)	c.556+1G>A	.	.	.
8	Index/proband (F)	c.C811T: p.R271X	NC_000018.10:g.667547T>A (LCR)	0.0047	*TYMS*, intron 3

U/A – unaffected; MAF- Minor allele frequency; LCR-Low complexity region; M – male; F- female.

Figure S1: Sanger traces of *TYMS* coding sequence from control and proband's whole blood DNA and cDNA

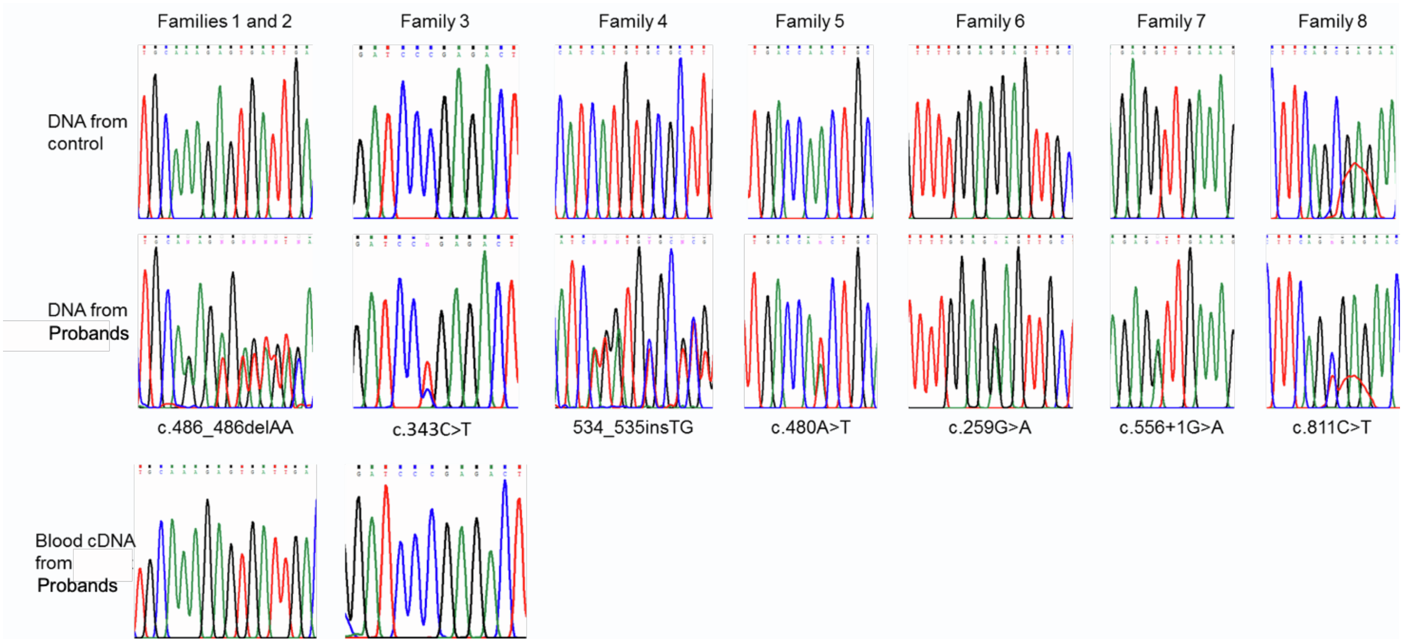

Representative Sanger traces showing the pathogenic variants identified in this study compared to an unaffected sample. Where RNA samples were available from the probands, we show that the variants in Families 1-3 cause nonsense mediated decay of the allele carrying the *TYMS* variant.

Figure S2. Clustal Alignment of *TYMS* in different species

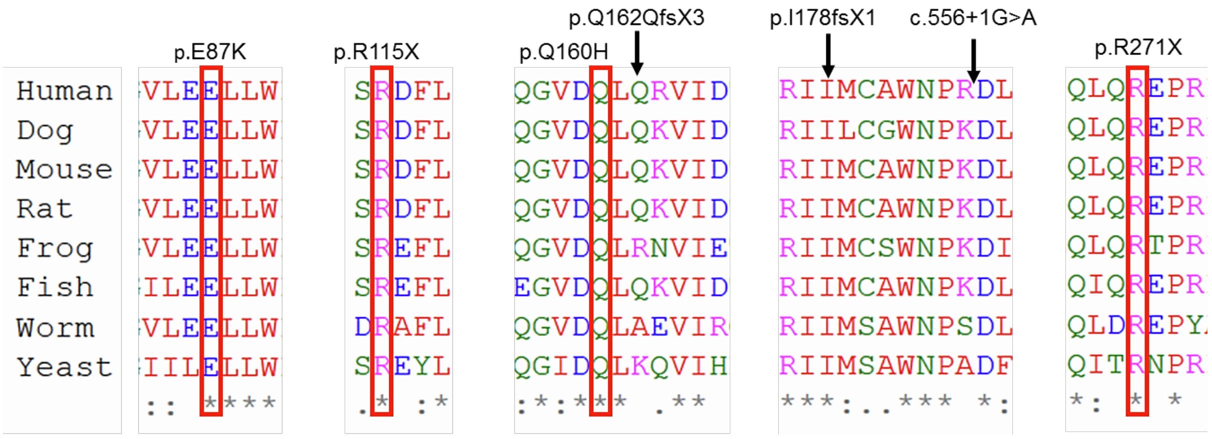

Clustal alignment of TYMS showing a high level of conservation between species. The position of the variants identified in this study are highlighted.

Figure S3: Telomere and telomerase associated growth defects in TYMS deficient cells of affected individuals

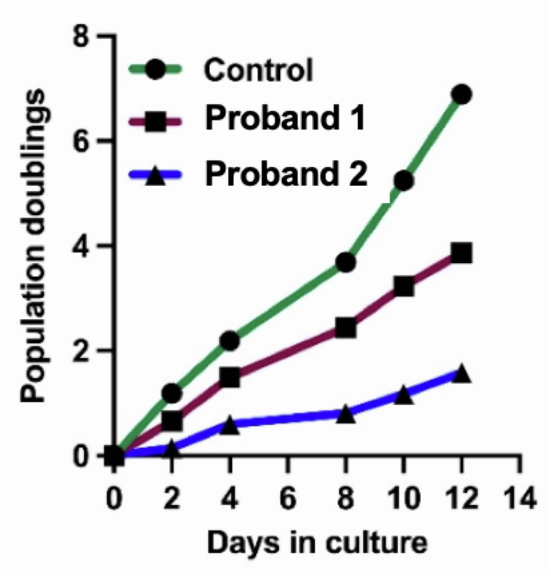

Population doubling rate of probands' cell lines (1 and 2) compared to control.

Figure S4: Assessment of DNA repair proteins in TYMS deficient individual cells along with control cells

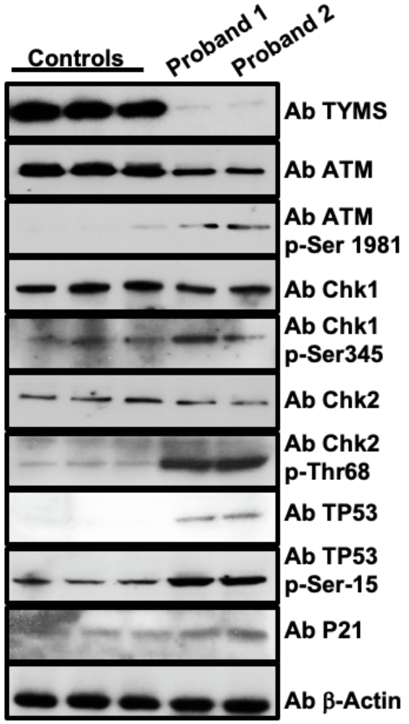

Immunoblotting of several key proteins involved in telomere maintenance and DNA damage response pathway. Antibody against β-Actin is used to determine the loading control.

Figure S5: Post transcriptional fate of *TYMS* is controlled by *ENOSF1*

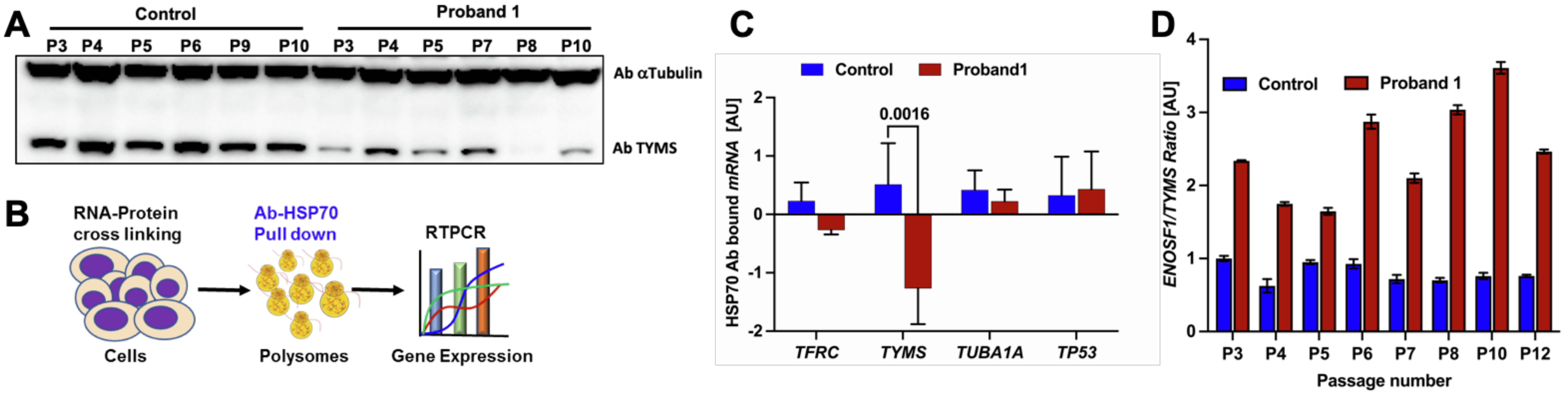

A) Reduced TYMS protein levels in proband samples over time at different passages. Antibody against α-tubulin is used to determine loading control.

B) Schematic diagram of crosslinking and immunoprecipitation of translating ribosome with affinity beads that are covalently attached with anti-Hsp70 antibodies. The crosslinked and anti-Hsp70 immunoprecipitated mRNA complexes are subsequently eluted and analysed by qPCR.

C) qPCR analysis shows a significant decrease in *TYMS* mRNA bound to the polysome when compared to input, while TP53 binding to polysome is increased in the proband samples. Bars represent the median of relative expression ratio.

D) *ENOSF1* transcript levels relative to *TYMS* expression in control and proband cells with increasing passage number.

Figure S6: Endogenous TYMS rescue and RNA-RNA interaction prediction between *TYMS* and *ENOSF1*

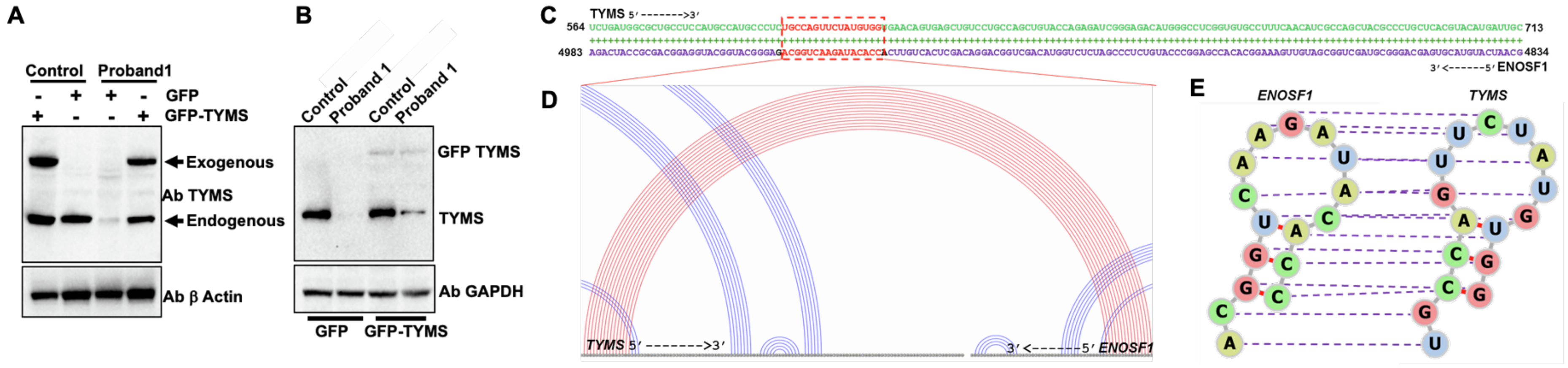

(A and B) Immunoblotting of TYMS protein in control and proband cells in two different transduction experiments at passage 3 (A) and passage 8 (B) transduced with lentivirus particles encoding GFP alone or GFP tagged *TYMS* cDNA. Antibody against β-Actin and α-tubulin is used to determine loading control. (C and D) IntaRNA verison 2.4.1 was used for prediction of RNA sequences involved in mediating RNA-RNA interaction between *TYMS* and *ENOSF1*. The red highlighted region in the sequence shows putative and accurate interaction region involved between *TYMS* and *ENOSF1* using RactIP. The red arched lines indicate intergenic interactions between two different genes, in this proband *TYMS* and *ENOSF1*. The blue arched lines indicated intragenic interaction within the gene.

Figure S7: Clinical features of affected individuals with biallelic *DUT* variants (NM_001025248.2)

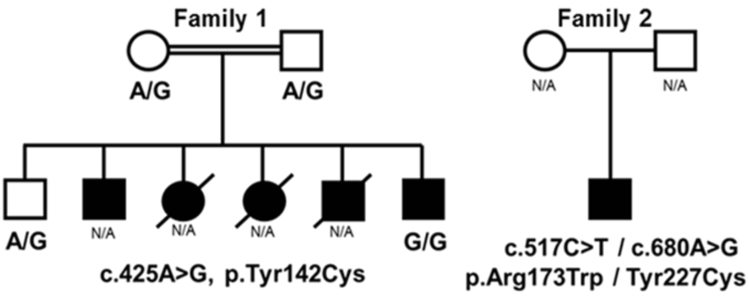

Features of individuals with biallelic *DUT* variants

Family	1	2
**Feature**		
Gender	M	M
Country/ethnic origin	Sudan	UK-Scotland
Age at investigation (years)	5	16
Parents first cousins/related	Y	N
Skin pigmentation abnormalities	Y	Y
Nail dystrophy	N	Y[c]
Leucoplakia	N	N
Hair loss/thin eye lashes	N	N
Haematological abnormalities	Y[a]	Y[d]
Immune defects	N	Y[d]
Learning/developmental difficulties	N	N
Microcephaly	N	N
Other features	Y[b]	Y[e]

Family 1 - (a) Investigations at age 5 years showed pancytopenia, hypocellular bone marrow with dyserythropoiesis and normal karyotype. This individual's history and clinical course have been documented in the European Journal of Haematology (1998; 60: 209-212). He was stable on oxymetholone and granulocyte colony stimulating factor therapy for several years; (b) developed insulin dependent diabetes mellitus while on oxymetholone therapy, at age 10 yrs progressed to myelodysplasia with monosomy 7. His family history was significant, 3 older siblings had died of bone marrow failure associated with diabetes mellitus in Sudan.

Family 2 – (c) many toenails affected; (d) at age 16 years was blood transfusion dependent and lymphopenic. Bone marrow was hypercellular exhibiting marked dyserythropoiesis and associated with splenomegaly; (e) Abnormal facies, small jaw, overcrowded teeth, growth restriction, short stature and hypogonadism. F = female, M = male; Y = yes; N = normal/no
